# U.S. consumer acceptance of whole grains, local grains, and alternative grain networks: a scoping review

**DOI:** 10.3389/fnut.2026.1729260

**Published:** 2026-02-13

**Authors:** Rebekah Schulz, Hikaru Peterson, Hallie Lundquist, Megan Kocher, Len Marquart

**Affiliations:** University of Minnesota Twin Cities, St. Paul, MN, United States

**Keywords:** alternative grain networks, consumer acceptance, consumers, local grain economies, local grains, regional grain networks, whole grains

## Abstract

**Introduction:**

Whole grains, including pseudocereals, provide a dual benefit: they support human health and disease prevention and strengthen agronomic and ecosystem functions through crop rotations. Despite numerous epidemiological and intervention studies indicating their health promotion and disease prevention benefits, however, only one percent of U.S. children and eight percent of U.S. adults meet actual whole grain intake recommendations. Recently, alternative grain networks as a type of grain system are emerging in the U.S., with a vision of offering local grain products to consumers not only for their benefit but also for that of the entire grain value chain.

**Methods:**

The aim of this scoping review is to map existing literature on U.S. consumer acceptance of whole grains, local grains, and alternative grain networks, define knowledge gaps, and make recommendations for future research. Following PRISMA-ScR guidelines, we systematically screened 14,155 articles, 32 of which met our inclusion criteria.

**Results and discussion:**

The included studies were methodologically heterogeneous, spanning randomized control trials, surveys, observational studies, and qualitative methodologies. The studies examined whole grains, bread, organic grain products, local grain products, and specific grain products. Due to most of the included studies’ focus on whole grains, the main focus of the scoping review is on whole grains. Collectively, the studies most frequently reported on the following key factors impacting consumer acceptance of whole grains: taste, price, knowledge, familiarity, attitude, and income/food insecurity. Many of the identified barriers and drivers to whole grain intake were similar to those in most other countries. Three articles examined consumer acceptance of alternative grain networks and local grain products. These articles, all using a qualitative, case study methodology, indicated that a select segment of consumers are interested in these systems and products, without quantitative measurement of consumer acceptance. The results of this scoping review suggest that educational campaigns such as those focused on whole grain identification, whole and local grain health and environmental benefits, preparation techniques and recipes, community cooking classes and sampling events, and policy and research advances along the grain supply chain could aid in promoting consumer acceptance of whole grains, local grains, and alternative grain networks.

## Introduction

Food systems are faced with a multitude of challenges in the twenty-first century, including the impacts of climate change on the content and quality of the food supply, biodiversity loss, the contribution of dietary patterns to environmental degradation, embedded forced labor, and the “triple burden” of malnutrition, a term which includes overnutrition (overweight, obesity), undernutrition (wasting, stunting), and nutritional deficiencies (1–3). An enhanced small grain sector, understood as one which prioritizes whole grains (WG) sourced locally, can help address these food systems challenges, as small grains play a well-researched and documented role through their agronomic and ecosystem benefits and their contribution to a healthy diet and disease prevention in their WG form (4).

Most small grains are the edible seeds from the grass family *Poaceae*, including cereals such as wheat, oats, barley, rye, emmer, einkorn, sorghum, wild rice, and millet, and pseudocereals including buckwheat, amaranth, and quinoa (5). While there is extensive biodiversity of grains available to growers, both between and within species, both agriculture and nutrition are dominated by low-diversity systems. For example, in the U.S. monoculture system, wheat represents the third largest planted acreage after corn and soy. In addition, there are commonly only a handful of varieties predominantly grown on the landscape, such as in Minnesota, where a little over half of the planted acreage consisted of three varieties in 2024 (personal correspondence, MN Wheat Breeder). As follows, alternative small grains like oats, rye, and millet make up a marginal percentage of U.S. planted acres (6). As a result, commodity grain systems provide us with most of our grain products, and are characterized by centralized geographical growing locations, consolidated intermediate processing owned by multinational corporations, and refined grain (RG) products as the target product endpoint (7, 8).

A growing body of research indicates that including a greater diversity of small grains in multi-year, corn- and soy-dominant rotations yields both agronomic and ecosystem benefits such as improved overall crop yield, soil health, and water quality parameters (9–12). Furthermore, adding small grains to crop rotations can diversify farmer income through new grain markets, including offering consumers local, value-added, small grains, a largely overlooked though burgeoning food in the local foods movement characterized by any small grain that is processed and used as food or beverage for human consumption (13, 14). As follows, alternative grain networks (AGNs), a type of local or regional food system that exist as alternatives to the conventional food system, are gaining traction on the landscape, establishing new markets for farmers, incentivizing intermediate processing infrastructure, benefiting local economies and communities, and offering value added, nutritious and delicious grain products to consumers (15). While the terms “local” and “regional” can be confusing due to the vague nature and multiplicity of definitions existing for these terms, we will use them interchangeably, and describe them as meaning grains, infrastructure, and markets offered within the region where a consumer lives, be that a state or a multi-state area (16). Similarly, while “small grains” is the technical term, we will use “small grains” and “grains” interchangeably.

Value-added, small grain production systems, whether commodity or AGNs, have the potential to offer a variety of grain products in a variety of forms, and it is well-established that WG, grains that include the outer bran, starchy endosperm, and germ of the kernel in their original amounts are beneficial for human health due to their bioactive properties, including vitamins such as folate and phytochemicals such as lignans, and fiber (17, 18). WG consumption is known to decrease risk of cardiovascular disease, some cancers, weight gain and type 2 diabetes, along with benefiting the gut microbiome, with improvements in physiological measures (19–22). Conversely, a low intake of WGs is the second most significant contributor to global diet-related mortality rates (following high sodium intake), and the first when looking at disability adjusted life years (DALYS) (23). A modest 0.25 oz eq/day increase in WG intake would result in a U.S. one-year-CVD healthcare savings cost of $2.4B ($0.6B to $4.3B) according to Murphy and Schmier (24). However, while a large diversity of grains exists both within and among species, potentially offering a diversity of WG options, Americans consume mostly refined grains (RG), with most breads and pastas sourced from a handful of varieties of wheat, and tortillas from one to two varieties of corn (25). WG undergo milling and extraction processes to create RG, sifting off the bran and germ and leaving the starchy endosperm. Making RG took off around the 1870s in the U.S. with the introduction of roller milling due to the commercial baking industry’s desire to standardize, scale, and create white flour and cereal products, but these products are well known to be nutritionally inferior to WG (8, 26). As high-glycemic foods, RG are associated with adverse metabolic effects, including increased risk for cardiovascular disease, metabolic dysfunction, and weight gain (27, 28) as well as poor nutritional status and a variety of diseases that began in the early 1900s due to refinement.

Due to the biochemical nutritional ideology of the 1940s in the U.S. (29), these deficiencies were viewed to be easily remedied, and the nutrient-stripped flour was subjected to flour fortification as a way to “fortify” the grain with the micronutrients removed during the milling process including thiamine to prevent Beriberi, riboflavin to enhance vitamin B6 and niacin utilization, niacin to prevent pellagra, and iron to address anemia (25, 29–31). Folic acid fortification began in 1998 to prevent neural tube defects in the developing babies of pregnant women (30, 32), with current debate on the impacts of the synthetic form of folic acid fortification and its health outcomes (25). While enrichment is not mandated, any grain or bread product labeled as “enriched” is required to meet the Federal Drug Administration’s (FDA) standard levels of these micronutrients (33). While the amounts of these micronutrients that are added back in are greater than the original WG, the enriched refined flour does not consider other nutrients lost such as magnesium, potassium, fiber, and protein (34). As a result, Metcalfe (25) writes that enriched RG products have a different nutrient profile and grain structure to that of WG including being devoid of fiber and containing nutrients existing outside the original synergistic complex.

In 2005, the Dietary Guidelines made the first WG recommendation to consume at least three servings of WG daily (1 serving = 1 ounce equivalent (oz. eq.) = 16 grams (g)) with the statement, “make half your grains whole” while encouraging the limitation of refined grains to three servings or less (35). However, most U.S. consumer intake of WG is insufficient. According to the 2020 Dietary Guidelines for Americans (DGAs), only 2% of American adults meet the recommended intake of = 3 servings of WG a day while 74% exceed the RG recommendations (36). In addition, more than 94% of Americans have an inadequate intake of dietary fiber, leading to what is deemed “the fiber gap” in the U.S. and worldwide (25). The removal of the fiber-rich bran in industrial wheat flour is proposed as a main contributor to this gap (25, 37, 38) along with the low consumption of higher fiber grains such as barley and rye in typical American diets. Switching current grain consumption from RG to WG could have astronomical impacts for improving health outcomes related to the fiber gap, as common wheat alone makes up one in five calories worldwide and up to 50% calories in some places (39). As a result, researchers, industry leaders, practitioners, and policy makers have been working to find ways to increase WG consumer acceptance and consumption in various populations, whether through commodity or AGNs.

Many national and international efforts exist to promote WG intake, including conferences such as the International Whole Grain Summit, and groups such as the Whole Grains Council (U.S.), the Danish Whole Grain Partnership (DWGP), and the Australian Grains and Legumes Nutrition Council (GLNC). While local grains are not inherently interchangeable with WG as the milling and sifting practices of small mills varies, AGNs inherently value local, value-added, small grain products, and are supported regionally by non-profit organizations such as the Artisan Grain Collaborative (AGC), the Washington State University (WSU) Breadlab, the Maine Grain Alliance, the Colorado Grain Chain, and the Glynwood Center for Regional Food and Farming. As a result, these AGNs have great potential to lead the charge on promoting WG that not only nourish humans, but also local economies and soil through the values inherent to these systems, as exemplified by the WSU Breadlab’s mission to see farmers, millers, bakers, and consumers breed, grow, and eat the whole grain as a world-wide habit.

Several studies have examined barriers and drivers to consumer acceptance of WG specific to individual countries including the U.S. (40, 41), Northern Ireland (42), Australia (43, 44), Northern Italy (45); the UK (46); and Finland (47), as well as reviews synthesizing global data (48, 49) identifying key indicators such as taste, convenience, and perception. In addition, Suthers, Broom and Beck (49) conducted a systematic review summarizing key strategies of successful public health WG campaigns. However, despite these efforts, along with isolated studies and marketing surveys, there is no comprehensive scoping review mapping U.S. consumer acceptance of WG, local grains, or consumer engagement with AGNs to determine the state of the literature, research gaps, and future research recommendations. While Meynier et al. (48) included the U.S. in their study, their review utilized general literature review methods and they did not report how they collected or analyzed the data.

To provide U.S. specific consumer insights, this scoping review sought to identify all pertinent studies that examine U.S. consumer acceptance of grains to map existing evidence, identify key themes, and point out knowledge gaps. Such a review will allow for an examination of the breadth of factors impacting U.S. consumer acceptance of grains and alternative grain networks, allowing for comparison to other countries to determine if there is anything particularly novel about the U.S. market, and to aid researchers, practitioners, nutritionists, and public health officials in tailoring communication strategies and educational campaigns to U.S. consumers to increase WG intake. While alternative grain networks (AGNs) do not necessarily explicitly focus on WG, they tend to be oriented to encouraging WG due to the inherent values such as nutrition, health, and craft, embedded within these systems. As epidemiological and nutritional studies are most often interested in WG intake as opposed to general grain intake, we utilize this language throughout the rest of the scoping review.

We searched five subject specific databases with a publication timeframe of the last fifteen years for relevance to current trends. Our primary research question was as follows: “What are the key factors influencing U.S. consumers’ acceptance of grains including reasons that motivate or deter consumption such as nutrition, price, dietary practices, and taste?” (RQ1) Our two secondary research questions looked to understand consumer acceptance of local/regional grain products and consumer engagement with types of grain systems, such as AGNs. These questions are as follows: “What are the key factors influencing U.S. consumers’ acceptance of local/regional grain products?” (RQ2A), and “How do U.S. consumers engage with grains including through the commodity grain system or alternative grain networks (AGNs)?” (RQ2B)

## Methods

### Protocol and registration

A protocol for this review was developed by the authors and reported in accordance with the PRISMA-P guidelines for systematic review protocols (50) with adaptations made to match scoping review criteria. The protocol was registered prior to data collection in the Open Science Framework (51). This scoping review was conducted in accordance with the JBI Manual for Evidence Synthesis, Chapter 10 (52) and is reported according to the PRISMA extension for scoping reviews (PRISMA-ScR) standards (53).

### Eligibility criteria

Studies were included in this review if they addressed the key elements of the research question dealing with grain consumption patterns of U.S. consumers. In the interest of relevance to current practices, articles were only included if they were published in 2013 or later. We set this publication window to find the most up-to-date evidence on consumer acceptance due to the rapidly changing nature of consumer trends and product innovation. Only English-language articles were included due to the focus on U.S. consumers, and article types were limited to dissertations, theses, and peer-reviewed journal articles. Studies were not included if they were secondary or tertiary sources or if they lacked sufficient information to understand the link between grain and U.S. consumers. Additionally, studies were excluded if their main focus was on the following:

- producer interactions
- non-food grains
- grain-based or grain-additive products from a consumer sensory acceptability, food product acceptability or modification, food quality, ingredient formulation study, and/or from a food science formulaic lens
- Specific health outcomes or interventions related to grain consumption
- Market analysis of grains
- Food-waste of grains

### Information sources and search

Searches were conducted across five databases: CAB Abstracts (Ovid), Agricola (Ovid), Scopus (Elsevier), EconLit (Proquest), and Dissertations and Theses Global (Proquest). Additionally, backward and forward citation searches were performed on all included articles in Scopus. Searches were developed by a science librarian (MK) in consultation with subject matter experts to capture articles discussing small grains, consumers, and their interactions. Searches tested against a set of exemplar articles. The full search strategy for each database can be viewed in Supplementary material. All searches limited publication dates to 2013 or later. Initial searches were run in all databases on October 24, 2024. Backward and forward citation searches for included articles were run on November 21, 2024.

### Selection of sources of evidence

All retrieved search results were uploaded to Covidence for deduplication and screening. Screening was done in two phases: title/abstract screening and full-text screening. In each phase, two independent reviewers screened each record and only those receiving two positive votes were moved on to the next round. In the case of opposing votes, a consensus process was used to make decisions via a third reviewer and discussion between the two primary reviewers.

### Data charting process

We utilized the data extraction feature on Covidence to chart the data in provided tables. The form was semi-calibrated, and we added our own criteria for each type of data that we wanted to extract. Data charting was done independently by one reviewer. There was no need to obtain or confirm data from investigators.

### Data items

We extracted data on article characteristics (e.g. publisher, authors, country of origin, type of study), demographics of included participants, types of grains studied, barriers and motivators to consumer acceptance, the outcomes of interventions, outcome variables (e.g. attitude, belief, choice, etc.), and key findings.

### Synthesis of results

The extracted data tables were downloaded from Covidence and re-formatted into a spreadsheet. One reviewer descriptively analyzed and grouped this data based on similar key findings in consumer acceptance as well as whether alternative grain networks were mentioned. The reviewer created categories for each key finding and included associated articles and findings in each section of the results. For example, definitions or groupings of variables affecting consumer acceptance including categories such as intrinsic product-related attributes (taste, color, aroma, texture, chemical and physical composition), extrinsic product related attributes (information, packaging, labeling, nutrition claims), personal state factors (psychological components, physiological needs, habits), cognitive factors (attitude, liking, preference), and sociocultural factors (culture, income, policy) (54). While product related attributes are often categorized into intrinsic (characteristics of the food itself such as physical composition, taste, smell, appearance, and texture) and extrinsic (characteristics that are related to but not physically part of the product such as labeling, packaging, nutrition claims, and price) attributes (55) based on the “cue utilization theory” (56), a theory that suggests that consumers take cues on whether to purchase a product based on the product’s intrinsic and extrinsic qualities, this review combined all product related attributes together. Due to a recent network analysis by Hoffman et al. (55) which calls for research to focus on the interplay between extrinsic (e.g., price, brand and country of origin) and intrinsic (e.g., taste and health) attributes as well as the food environment on consumer acceptance so as to develop a more comprehensive picture of the factors involved, we developed the “environmental factors” category to group extrinsic factors from our studies that were not otherwise accounted for.

## Results

**FIGURE 1:**
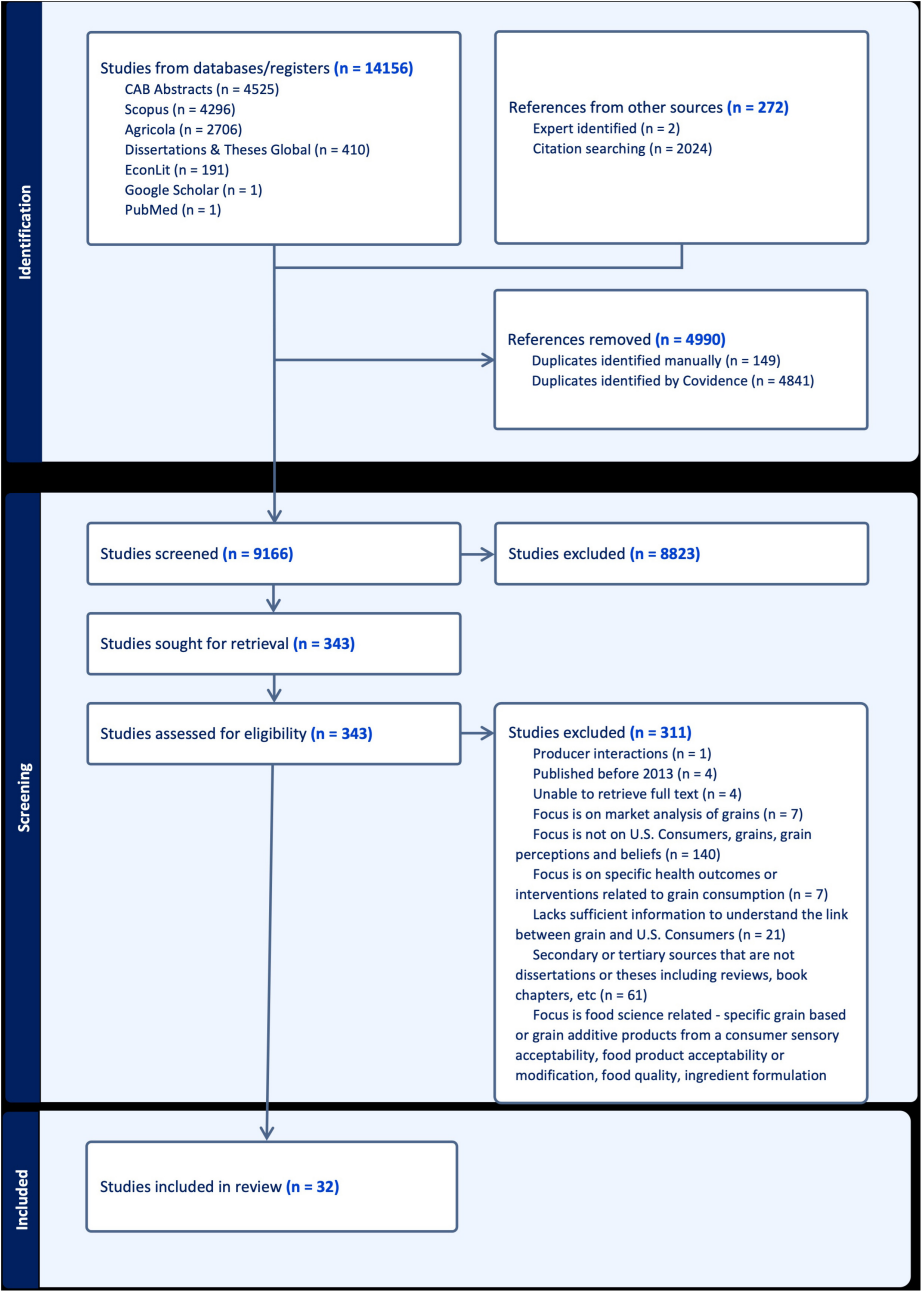
PRISMA diagram reporting scoping review process.

The Preferred Reporting Items for Systematic Reviews and Meta Analyses (PRISMA) diagram outlines the search strategy and screening process (Figure 1). After the initial search, we imported 14,155 articles into Covidence and screened for duplicates, resulting in 9,165 studies. The initial screening stage resulted in excluding 8,823 of these studies due to irrelevance, with 341 full-text articles remaining. The full-text screening removed 310 studies for various reasons with 31 remaining. One additional article was retrieved through hand searching, bringing the total of included articles to 32. We included these in the data extraction phase and documented them in this scoping review.

### General trends

While our first research question looked at grains as a general category, most studies (*n* = 19) included in the review reported on overall behaviors and attitudes toward WG at large, including a similar variety of products such as bread, crackers, cereal, pasta, cookies, and cakes, but did not attach specific findings to specific products. As a result, this review and the following results section mainly focuses on WG. Two studies utilizing National Health and Nutrition Examination Survey (NHANES) data examined “grains” as a broad category, and only inferred WG intake based on carbohydrate, fiber, and energy density data (57, 58). When reporting on these studies (*n* = 2), we combined them with the other WG studies for the purpose of the review synthesis. After WG, bread was mentioned the most (7), followed by organic wheat products (3) and local grain products (3); specific grain products such as WG pizza or a high-antioxidant sweet corn were only addressed in one to two studies each, which we grouped together in a category termed “specific grain products” for analysis (*n* = 8) (Table 1). We based our grain product categories on the National Health and Nutrition Examination Survey (NHANES) (53), and created some additional categories based on themes identified in the literature. Three studies attended to consumer engagement with local grains and alternative grain networks (AGNs) through qualitative case studies examining local bread, flour, and grains. Three studies focused on organic wheat products (including bread, cookies, and croissants) to examine consumer valuation of these products. Finally, ten studies were interventions focusing on improving consumer acceptance of WG and other grain products. We summarized these studies in Tables 2, 3. There were no studies covering the following categories: snack/meal bars, savory snacks/crackers, Mexican mixed dishes (i.e., burritos, tacos, nachos), soups, and infant foods.

**TABLE 1:** Grain product categories based on National Health and Nutrition Examination Survey (NHANES) categories documented in scoping review with references.

Grain product categories
Whole grains (*n* = 19) •Lenk et al. (83), Curtis et al. (84), Tester et al. (82), Onufrak et al. (145), Dunford et al. (81), Janhs et al. (67), Nicklas et al. (66), Leak et al. (78), Combest and Warren (60), Chea and Mobley (69), Magaliset al. (70), Chea and Mobley, (68), De Leon et al. (73), Samuel et al. (64), Wongprawmas et al. (62), De Leon et al. (72), Arts et al. (85), MacNab et al. (79), and Yao et al. (77)
Breads (*n* = 7) •Mars (87), Baker and Russell (86), Meyer et al. (88), Drugova and Curtis (74), Drugova et al. (75), Lin et al. (59), and Samuel et al. (64)
Organic grain products (*n* = 3) •Drugova et al. (75), Drugova and Curtis (74), Lin et al. (59)
Local grain products (*n* = 3) •Baker and Russell (86), Mars (87), Meyers et al. (88)
Specific grain products (*n* = 8) •Ready-to-eat-cereals: ready-to-eat-cereals (63) Cakes, cookies, pies, pastries, bars: all-purpose flour and white whole wheat flour shortened cakes, muffins and yeast bread (64) Cooked grains and cereals: hybrid rice and whole grain pasta (62, 76)Pizza, burgers, sandwiches: whole grain pizza (71) Other mixed dishes: chicken-and-whole-grain-pasta dish (61) “Other” grains*: Brewer’s spent grains baked goods (60), red, hi-A corn variety (65).

The “specific grain products” category lists the NHANES category followed by the items within that category studied in each respective paper. * Category created based on themes in literature review.

**TABLE 2:** Characteristics of included studies.

References	Research design	Sample size	Study population	Grain product	Key factors	Outcome variable(s)
Baker and Russell (86)	Case study	n/a	NY Farmer, NY Millers (3), NY Artisan Baker, NY consumers	Variety of local grains and local grain products (local and organic small grains, flour, and artisanal bread)	Price, Accessibility, Environment (Community Program)	Purchasing behavior
Chea and Mobley (68)	Mixed methods: survey and interview	Nutrition educators (*n* = 9); low income adults (n = 98)	EFNEP Nutrition educators working with low-income adults; low-income parents or guardians with at least one child between 3 and 10 yo; all; > 18 y.o.	Whole grain products	Taste, Price, Familiarity, Perceptions, Knowledge, Sociodemographic (Low-Income)	Consumer perceptions; consumer preferences
Chea and Mobley (69)	Mixed methods: survey and interview	169 (survey); subset 60 (interview)	Low-income adults; > 18 y.o.	Whole grain products	Taste, Price, Knowledge, Attitudes, Sociodemographic (Age, Country of Origin, Education Level, Low-Income)	Consumer perceptions
Combest and Warren (60)	Focus group	37	University students; 18–45 y.o.	Whole grains, Brewer’s Spent Grains (cookies, sweet breakfast breads, granola bars)	Taste, Price, Familiarity, Perception, Knowledge, Attitudes, Convenience, Sociodemographic (Age)	Barriers and drivers to consumption; consumer perceptions; sensory attributes
Curtis et al. (84)	Case study	33	14 families from four separate Utah CSA programs	Grains (generic)	Environment (Community program)	Purchasing behavior
Drugova et al. (74)	Choice experiment and survey	1,009	Adults; > 18 y.o.	Organic bread and cookies	Taste, Price, Labeling, Perceptions, Familiarity, Preference	WTP, Consumer preference, motivations and barriers
Dunford et al. (81)	Longitudinal study (Nielsen Homescan 2018 data)	61,367 households	Adults; > 18 y.o.	Whole grain products	Sociodemographic (Low-income, Household size, Country of Origin)	Purchasing behavior
Hicks-Roof et al. (61)	Sensory evaluation	664	Adults; ≥ 18 y.o.	Sorghum mixed dish and rice mixed dish	Taste, Preference, Sociodemographic (Country of Origin)	Acceptability, consumer preference
Jahns et al. (67)	Nominal group technique	51	American Indian Fifth grade children (ancillary study to *HEALTH* study)	Whole grain products	Preference, Taste, Environment (Home)	Consumer preferences
Johnson et al. (65)	Contingent valuation and survey	1,037	Adults; > 18 y.o.	Red, Hi-A? (high-antioxidant) corn variety	Price, Taste, Labeling, Preference, Sociodemographic (Age), Built environment (Food outlet availability)	WTP, Consumer perceptions
Lenk et al. (83)	Interview	661	Customers of small and non-traditional food stores (corner stores); Adults; > 18 y.o.	Whole grain products	Sociodemographic (Education, Country of Origin, Gender)	Shopping pattern
Lin et al. (59)	Choice experiment	263	Adults; > 18 y.o.; consumed bread in last 3 months	Conventional WG bread, organic WG bread, carbon labeled WG bread	Price, Knowledge, Sociodemographic (Education, Country of Origin)	WTP, Knowledge, Consumer preferences
Magalis et al. (70)	Survey	69	College students; > 18 y.o	Whole grain products	Taste, Price, Preference, Knowledge, Attitude,	Attitude, knowledge, liking/acceptability
Mars (87)	Case study	10	Participants in Arizona local grain network (e.g., baker, consumers)	Local heritage grains: organic Sonoran wheat; local artisan bread	Price, Taste, Preference, Attitude, Knowledge, Environment (Community Program)	Consumer preference
Meyer et al. (88)	Case study	Not noted	Participants of South Tyrol, Italy, and Colorado, United States local grain network	Local artisan bread, flour, and grain products	Taste, Price, Knowledge	Knowledge
Nicklas et al. (66)	Nominal group technique	281 (Adult caregivers); 321 children	National sample of 5th grade children and unrelated adult caregivers (part of *HEALTH* study)	Whole grain products	Taste, Price, Labeling, Knowledge, Convenience	Barriers and drivers of consumption
Onufrak et al. (145)	Epidemiological (NHANES)	25,549	Census representative US adults (NHANES 2005–2014); > 20 y.o.	Whole grain products	Environment (Food Outlet)	Barriers and drivers of consumption
Setiono et al. (57)	Epidemiological (NHANES)	4,065	Census representative adolescents (2011–2018 NHANES); 12–19 y.o.	Grains (inferred WG based on fiber and carbohydrate data)	Environment (Food Outlet)	Shopping pattern
Taylor et al. (58)	Epidemiological (NHANES)	20,363	Census representative US adults (2005–2012 NHANES); ≥ 20 y.o.	Grains (inferred WG based on energy density data)	Sociodemographic (Low-Income)	Dietary pattern
Tester et al. (82)	Epidemiological (NHANES)	3,265	Census representative adolescents (NHANES 2005–2012); 13–18 y.o.	Whole grain products	Sociodemographic (Low-Income)	Barriers and drivers of consumption
Tritt et al. (71)	Mixed methods: plate waste and sensory evaluation	394 (Child restaurant patrons); 120 (school children)	School children (Grades 3–5; 8–11 y.o.)	Whole grain pizza and refined grain pizza	Taste	Acceptability
Wongprawmas et al. (62)	Survey	499	U.S. college students; ≥ 17 y.o.	Whole grain pasta	Taste, Price, Perceptions, Attitudes	Attitude; Barriers and drivers of consumption

NY, New York; WTP, willingness-to-pay; y.o., years old.

**TABLE 3:** Characteristics of included intervention studies.

References	Research design	Sample size	Study population	Key factors	Grain product	Outcome variable(s)	Intervention type	Intervention details	Intervention outcome
Akaichi et al. (76)	Experimental auction	350	U.S. food shoppers; ≥ 18 y.o.	Perceptions, Preference, Attitude, Sociodemographic (gender, age, education, income)	Hybrid Rice; Conventional Rice	Willingness to pay	Information provision	Experimental auction; participants exposed to the following information though labeling: low greenhouse gas (GHG) emissions, food miles, and origin of food	Consumers are WTP a higher premium for hybrid rice labeled as lower GHG emissions; consumers don’t value food miles and origin of food attributes equally
Arts et al. (85)	Nutrition education intervention	98	College students	Information provision	Whole grain products	Total consumption	Information provision	6-week intervention; WG and low-fat dairy messages in campus dining halls and text messages to subsample (*n* = 26); prompts to choose WG given in dining halls; nutrition education booths for WG and low-fat dairy in one dining hall	WG intake increased from baseline to follow up (23 ± 31 to 31 ± 43 g) (*p* = 0.008) (35% increase in intake); POS messaging preferred messaging
De Leon et al. (72)	Randomized control trial	45	Healthy adults; ages 20–45	Taste, Attitude, Familiarity, Perceptions, Sociodemographic (gender)	Whole grain products	Acceptability	Exposure and familiarization	6 weeks; whole-grain group received whole grain products; refined-grain group received refined grain products	WG group participants increased intake from < 1 serving/day to 2.0–2.9 serving/d (*p* < 0.001) while there was no change for the RG; overall liking scores significantly improved for both the RG and WG groups for RG and WG; WG perceived as healthier by participants in both group (*p* < 0.001) and by women more than men (*p* = 0.032); all participants were willing to include RG and WG foods in diet at the end of the intervention than at baseline (*p* = 0.049)
De Leon et al. (73)	Randomized control trial	45	Healthy adults; ages 20–45	Taste, Attitude, Familiarity	Whole grain products	Attitude, consumer preferences, consumer behavior change	Exposure and familiarization	6 weeks; whole-grain group, refined-grain group	No significant difference between WG and RG groups in WG consumption; participants who originally preferred RG shifted to associate WG with good taste (*p* = 0.004) and in WG group (*p* = 0.032)
Dominick et al. (63)	Choice experiment	1,265 adults	Adults; > 18 y.o.	Knowledge, Price, Taste, Convenience, Sociodemographic (household size, food security)	Ready-to-eat-breakfast-cereal	Purchasing behavior, willingness to pay, consumer choice	Information provision	Random parameters logit model utilized to test WTP for nutritional elements of ready-to-eat-breakfast-cereal; nutritional information provided	Summary of nutritional highlights did not change valuation of healthier attributes
Drugova and Curtis (75)	Choice experiment	721	Adults; > 18 y.o.	Taste, Price, Preferences, Perceptions, Knowledge	Organic bread, organic cookies, organic croissants	Willingness to pay; preferences; barriers and drivers	Exposure and Familiarization; Information provision	Random parameters logit model utilized to test WTP for organic wheat products; provided information on organic labeling standards	Favorable taste assurances positively impact consumer acceptance and WTP; organic labeling standards information did not significantly impact consumer organic product perceptions or taste preferences
MacNab et al. (79)	Nutrition education intervention	139	Community residing adults = 60 y.o.	Knowledge, Familiarity	Whole grain products	Knowledge, total consumption, Liking/acceptability	Exposure and familiarization; Educational programming	Pilot study; 3, 1-hour sessions; WG education and discussion, hands-on activities, taste testing; pre and post questionnaires	Mean WG knowledge score increased 9% from pre to post (15.1 ± 4.9 to 21.6 ± 4.0); mean WG intake from pre to post (10.4 ± 4.5 g) to (11.3 ± 3.9 g)
Leak et al. (78)	Interview (following FoodRx intervention)	48	Low-income caregivers and prediabetic children dyads; caregivers (28–76); children (8–17); FoodRx study	Knowledge, Attitude	Whole grain products	Barriers and drivers of consumption; Consumer perceptions	Exposure and familiarization; Information provision	FoodRx pilot study (convenience sample of 60 obese, and prediabetic youth (8–17 years); received bi-weekly deliveries of whole grains and weekly delivers of vegetables for 16 weeks, qualitative exit interviews were given to youth caregivers	Participants had increased WG knowledge and positive attitude about WG and improved WG purchasing behaviors at the end of the intervention
Samuel et al. (64)	Sensory evaluation	102	College students; ≥ 18 y.o.	Taste, Familiarity	All-purpose flour and white whole wheat flour (WWWF) shortened cakes, muffins, and yeast bread	Acceptability	Exposure and Familiarization; Information provision	Untrained, volunteer consumer panelists participated in sensory evaluation of bakery products in cafeterias; two rounds of sensory evaluation: in round one, no information provided on products; in round two, nutritional benefits of whole grains and expected flavor and texture profiles were given prior to tasting	Familiarization with white whole flour resulted in WWWF products that did not score differently from all-purpose flour counterparts on appearance, taste, and overall acceptability (*p* < 0.05). Following familiarization with health benefits and expected flavor of WWWF products, WWWF products scored significantly higher for overall acceptability compared to WWWF products tested before the intervention (*p* < 0.05).
Yao et al. (77)		409	Food pantry clients who are responsible for meal preparation for family; control group (204), intervention group (205)	Perceptions, Knowledge	Chicken-and-whole-grain-pasta dish	Consumer perceptions	Exposure and Familiarization; Information provision	4-week quasi-experimental study; control group and nutrition education intervention group; intervention group tasted chicken-and-whole-grain mixed dish and given ingredients and invited to prepare recipe at home	Perception of WG consumption (*p* = 0.001) and self-efficacy in choosing and preparing WG foods significantly increased for intervention group.

POS, point-of-sale; WTP, willingness to pay; y.o., years old; WG, whole grain.

### Publication trends of reported studies

**FIGURE 2:**
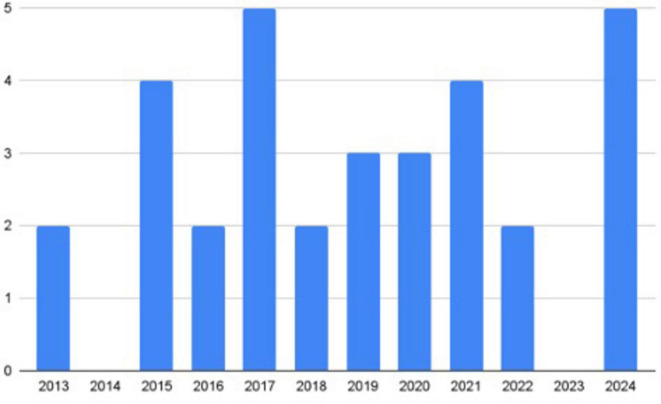
Publication years of included studies. No included studies were published in 2014 or 2023.

**FIGURE 3:**
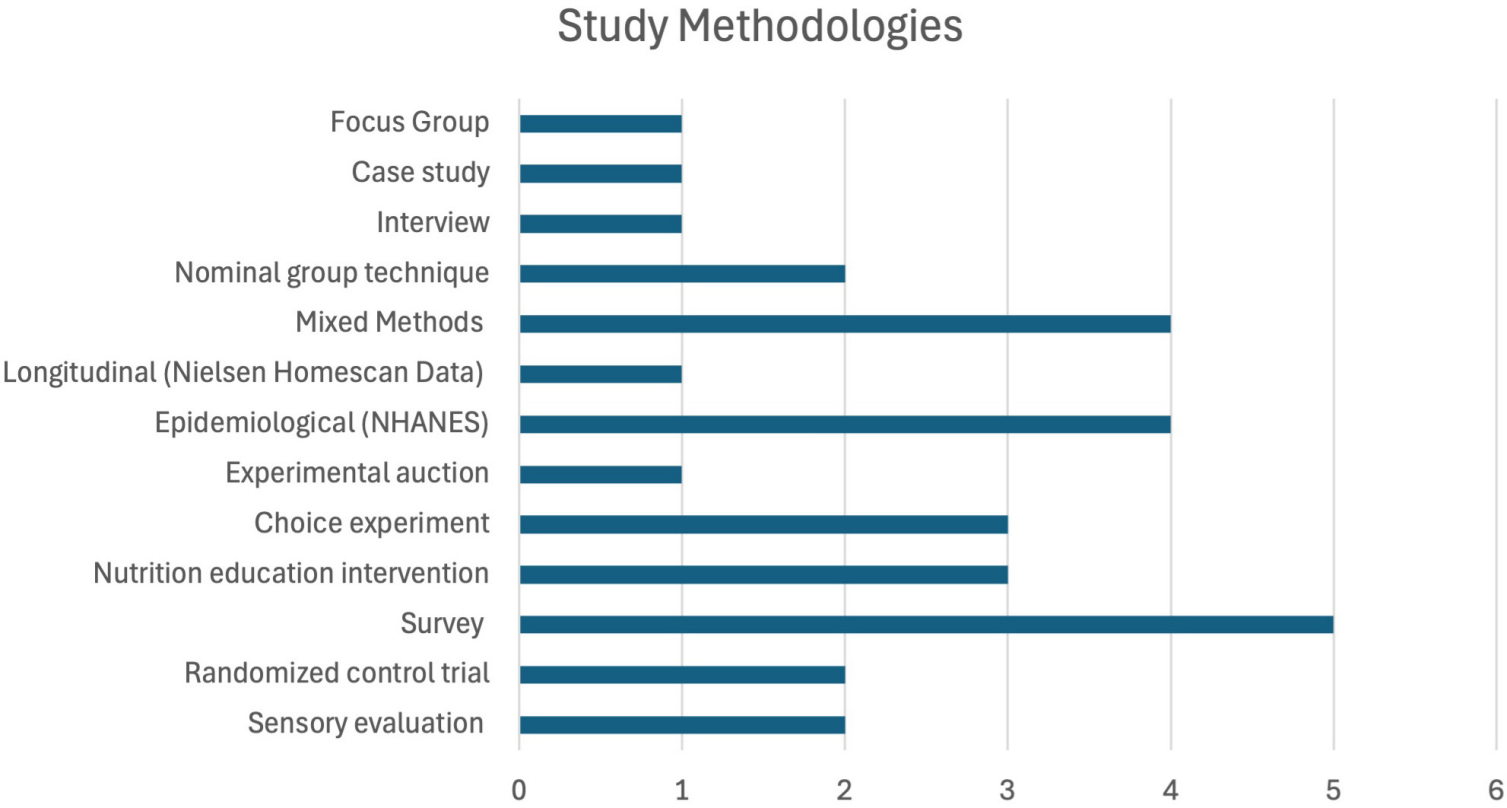
Methodologies of included studies. Counts are not exclusive across categories.

Between 2013 and 2024, five studies were published in 2017 and 2024, with four appearing in 2015 and 2021 and three in 2019 and 2020. Two studies were published respectively in all other years, except in 2014 or 2023, when there were none (Figure 2). Among the 32 studies, two each were published in *Current Developments in Nutrition, British Food Journal*; *International Journal of Environmental Research and Public Health*; *Journal of Agriculture, Food Systems, and Community Development*; *Journal of Food Distribution Research*; and *Public Health Nutrition.* The remaining studies were published in a range of different journals. Figure 3 documents the methods utilized by the various studies and Tables 1, 2 document the number of participants in each study.

### Consumer acceptance of whole grains

**FIGURE 4:**
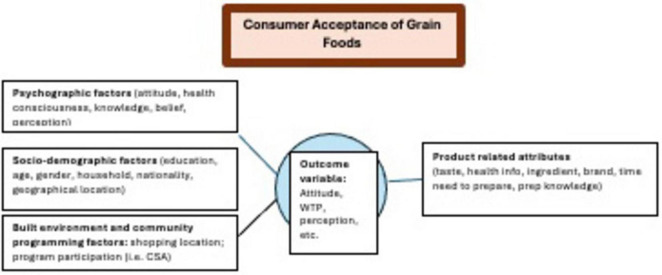
Four categories of key factors influencing consumer acceptance of grains foods with “outcome variable” as the proxy for measuring consumer acceptance. Adapted from Lu et al. (56), licensed under CC BY 4.0.

Based on our included studies, we placed the key factors influencing U.S. consumer acceptance of grain foods (RQ1) into the following four categories which were adapted from the literature: (1) product related attributes, (2) psychographic factors, (3) sociodemographic factors, and (4) environmental factors (Figure 4). The outcome variables reported in the studies were related to consumer behavior, attitudes, and willingness-to-pay for grain products. We also examined interventions reported in the included literature to increase consumer acceptance of grain foods with the following three types: exposure and familiarization, educational programing, and information provision. The extracted relevant key factors influencing consumer acceptance of grains foods were grouped and reported in Table 4. Most articles in the bread category [aside from Lin et al. (59)] are also reported in another category due to additional grain products documented in those articles.

**TABLE 4:** Key factors and interventions influencing consumer acceptance of grain foods, by grain product category.

	Whole grain Products	Bread	Organic wheat products	Local grain products	Specific grain products
**Product related attributes**					
Taste	x (66–70, 72, 73, 77)	x (64, 74, 75, 87, 88)	x (74, 75)	x (87, 88)	x (60–65, 71)
Price	x (66, 68–70)	x (59, 74, 75, 86–88)	x (74, 75)	x (86–88)	x (60, 62, 63, 65)
Labeling	x (66, 78)	x (74)	x (74)		x (65)
Convenience	x (66)				x (60, 63)
Accessibility		x (86)		x (86)	
	**Whole grain Products**	**Bread**	**Organic wheat products**	**Local grain products**	**Specific grain products**
**Psychographic factors**					
Perceptions	x (68, 69, 72)	x (74, 75)	x (74, 75)		x (60, 62, 76, 77)
Knowledge	x (66, 68, 69, 78, 79)	x (59, 75, 87, 88)	x (75)	x (87, 88)	x (60, 63, 77)
Familiarity	x (68, 72, 73, 79)	x (64, 74)	x (74)		x (60, 64)
Preference	x (67, 70, 73)	x (74, 75, 87)	x (74, 75)	x (87)	x (61, 65, 67, 76)
Attitude	x (69, 70, 72, 73, 78)	x (87)		x (87)	x (60, 62, 76)
	**Whole grain Products**	**Bread**	**Organic wheat products**	**Local grain products**	**Specific grain products**
**Sociodemographic factors**					
Income/food insecurity	x (58, 68, 69, 81, 82)				x (63, 76)
Country of origin and household size	x (69, 81, 83)	x (59)			x (61, 63, 76)
Gender	x (72, 83)				x (76)
Age	x (69)				x (60, 65, 76)
Education level	x (69, 83)	x (59)			x (76)
	**Whole grain Products**	**Bread**	**Organic wheat products**	**Local grain products**	**Specific grain products**
**Environmental factors**					
Food outlet	x (57, 145)				x (65)
Home	x (67)				x (67)
Community program	x (84)	x (86, 87)		x (86, 87)	
	**Whole grain Products**	**Bread**	**Organic wheat products**	**Local grain products**	**Specific grain products**
**Interventions**					
Exposure and familiarization	x (72, 73, 78, 79)		x (75)		x (64, 77)
Educational programing	x (79)				
Information provision	x (78, 85)		x (75)		x (63, 64, 76, 77)

Key factors are grouped by categories (product related attributes, psychographic factors, sociodemographic factors, and environmental factors) and interventions are categorized into three types: exposure and familiarization, educational programing, and information provision. Most articles in the bread category [aside from Lin et al. (59)]^1^ are also reported in another category due to additional grain products documented in those articles. The “specific grain products” category included the following products: ready-to-eat-cereal, baked goods, hybrid rice, whole grain pasta, whole grain pizza, a chicken-and-whole-grain-pasta dish, brewer’s spent grains baked goods, and a red, high antioxidant corn.

We will now discuss the specific findings for key factors influencing consumer acceptance of WG, bread, and specific grain products as reported in these studies, combining these categories into our WG synthesis and analysis as most of the products were WG. We will segment the findings by attribute and characteristic categories including interventions that utilized these factors to increase consumer engagement with WG:

1. *Product related attributes*
  a.    *Taste*

Taste preferences were consistently identified as a key factor influencing consumer acceptance across grain product categories including WGs, bread, organic specialty bakery products, and specific grain products such as brewer’s spent grain foods (60), a mixed sorghum dish (61), WG pasta (62), ready-to-eat cereal (63), white whole wheat flour baked goods (64), and a high antioxidant sweet corn (65) (Tables 2, 3). While taste is just one of many product sensory attributes, we report on taste as this was the factor primarily mentioned throughout the included studies.

Positive taste was a driver for WG consumption among Native-American fifth-graders in two studies utilizing the nominal group technique as part of the larger *HEALTH* study to examine factors influencing WG consumption, with poor taste as a barrier (66, 67). Two studies utilizing a mixed-methods approach (survey and interview) (68, 69) found that poor taste was perceived as a barrier for WG consumption in low-income adult participants. Magalis et al. (70) and Combest and Warren (60) utilized a survey and focus group respectively and found that university and college students who identified as WG consumers ranked WG bread as having similar sensory characteristics to RG bread and gave a higher liking for WG. This may be due to their familiarity already with WG. College students, however, liked the taste of the refined rice and pasta as compared to their WG counterparts (70). A comparative sensory analysis by Hicks-Roof et al. (61) found that college students ranked all sensory characteristics for a mixed-sorghum dish to be comparable to a mixed-rice dish except for texture/mouthfeel which was lower for the sorghum. Similarly, Tritt et al. (71) found that children liked the taste of WG pizza crust comparably to RG pizza crust, and college students found WG pasta to be somewhat tasty (62). Overall, these studies indicate that consumers display heterogeneity when it comes to WG taste. As mentioned below in the intervention section, there were a handful of studies largely showing that when accompanied with either familiarization through taste testing or information on health benefits and preparation, WG products had positive sensory acceptance and liking alongside the RG analogs (72, 73).

In organic specialty wheat bakery products (bread, cookies, croissants), two choice experiments looking to understand WTP for these products, indicated that as taste assurances improved, consumer acceptance increased regardless of their inclination toward organic foods (74, 75). Only consumers with a high inclination toward organics were willing to accept organic cookies, bread, and croissants of poorer or unknown taste (75), while those with less inclination for organics ranked taste as more important for cookies than bread (74). Freshness and taste were the two most important characteristics for organic bread and cookies.

b.     *Price*

Price is another product attribute with several studies examining the factors impacting consumers’ valuation for grain products, including grain attributes (i.e., WG, organic, local, carbon friendly), taste assurances, consumer segmentation, and product information. Regarding grain attributes, across caregivers of five-graders (66), low-income adults (68, 69), and University and college students (60, 70), WG were perceived to be more expensive than their RG counterparts. In contrast, college students were neutral on whether WG pasta was expensive, likely due to its availability in college cafeterias (62).

Regarding willingness-to-pay (WTP) for WG products, while a segment of a population always required a discount for organic cookies, bread, and croissants, other consumers were willing to pay a premium for these products, indicating high consumer segmentation (74, 75). In comparison to other attributes, consumers were more WTP a premium for organic bread over conventional bread or bread with a novel-carbon label in a choice experiment examining these attributes (59), and all consumers were WTP a premium for bread with the local label (74, 75). In an experimental auction, Akaichi et al. (76) found that consumers were WTP more for a lower GHG emission on hybrid rice, which could be further enhanced by the addition of local and lower food miles labels, as consumers did not see these labels as interchangeable.

While choice experiments revealed that WTP for organic wheat bread, cookies, and croissants (64) and a novel, high antioxidant sweet corn increases (58) as taste perception improves, WTP for ready-to-eat breakfast cereal (57) and organic specialty bakery products (64) does not improve with either a summary of nutritional benefits or education on organic labeling standards for organic, respectively. This may point to taste as being more of a motivator than information for WTP.

*c.*    *Labeling*

Product labels can play a role in influencing consumer acceptance of grains. Consumers utilized various labels and packaging cues to identify WG products, including reading the nutrition facts label (66) and ingredient list (70), using “whole grain” wording on the label, and looking at the color and appearance of the grain product (69).

Drugova and Curtis (75) and Drugova et al. (74) found that most consumers have low preference for the organic label on specialty bakery products unless they have a strong inclination for organic products, while most consumers prefer the local label on bread but not cookies. However, when both the local and organic label were present, the presence of the organic label decreased the utility of the local label for specialty bread on average, with only a segment of consumers viewing these labels as complementary. Additionally, those with a high value for organics valued the “natural” and “whole-grain” label on bread (74). Similarly, in a choice experiment examining consumer preference for a high antioxidant sweet corn, they found that preference for a local label resulted in higher WTP for the corn variety (65). These findings suggest that consumers display heterogeneity for different labels based on their consumer segment.

*d.*    *Convenience*

Only three included studies explicitly mentioned convenience, including a focus group in which college students reported purchasing WG products that can be eaten on-the-go more frequently and a choice experiment indicating that the majority of the Midwestern participants who reported being ready-to-eat breakfast cereal purchasers did so because of convenience (60, 63). Nicklas et al. (66) found that non-related caregivers of Native-American fifth-graders reported that having prepackaged WG or WG in foods that are easy to prepare and that they already eat would be a facilitator for WG intake.

1. *Psychographic factors*
  a.    *Perceptions*

Perceptions are a psychographic characteristic that influence consumer acceptance of grain foods. Combest and Warren (60) and De Leon et al. (72) both found that college students and adults perceived WG to be healthier than RG, respectively, and most consumers viewed organic wheat products as healthier than conventional (74). However, in a survey examining consumer acceptance of WG pasta, Wongprawmas et al. (62) found that while college-students perceived WG pasta to be more beneficial than RG and healthy, they did not see it as essential to include in the diet. This could indicate that although consumers perceive WG to be healthy, that perception alone is not enough to change consumer behavior. As mentioned in the intervention section below, several studies showed that interventions involving taste tests and exposure to WG can result in changed perception regarding their taste, attitudes toward WG, and perceived intake (72, 73, 77). When it comes to attributes of grain products, consumers did not perceive food miles and food origin as perfect substitutes for hybrid rice (76).

*b.*    *Knowledge*

Several studies indicate that general knowledge about WG identification and WG preparation is low. Less than half of low-income adults (69) and slightly more than half of college-students (70) correctly identified WG based on the ingredients list, with college-students unable to identify the differences between RG and WGs or the definition of WG foods (60), and the majority of low-income adults misinterpreting the WG message (make half your grains whole) (68). Chea and Mobley (68) found that while most low-income adults perceive WG dietary guideline messaging as easy to understand, they commonly misinterpreted the information. As a result, while consumers may perceive that their knowledge is adequate when it comes to WG identification, performing the task of WG identification indicates that most consumers lack the necessary knowledge for this task.

Three studies found that lack of skills on how to prepare WG at home and recipes for WG foods were barriers to consumer acceptance of WG in college students (70), unrelated caregivers of fifth-grade children (66) and caregivers of children with prediabetes and obesity (78). However, the type of information may matter, as two studies found that providing nutrition information and information on organic practices did not alter WTP for ready-to-eat-breakfast-cereal or organic specialty bakery products respectively (63, 75). Conversely, those participants who were familiar with certain types of environmental information such as the causes of climate change had a higher WTP for bread with a novel-carbon label compared to those participants who did not know this information (59). As mentioned in the intervention section below, some studies found that taste testing of WG resulted in increased ability to prepare and utilize them (77, 79). As a result, interventions such as information provision and tasting may differ in their impact on increasing consumer knowledge about WG.

*c.*    *Familiarity*

Six studies examined the impact of familiarity on consumer acceptance of grain products. Lack of familiarity was a barrier for WG consumption in low-income adults (68), purchasing of organic bread and cookies in the average consumer (74), and consumption of brewer’s spent grain products in college students (60). In two randomized control trials with a duration of 6-weeks, in which one group received WG products and the other group received RG products, exposure to WG over a 6-week period resulted in significantly improved ratings of liking, sensory profile, and willingness to include WG in the diet in consumers (72), as well as an improved positive implicit association for WG in those initially preferring RG (73). However, improvements in sensory acceptability for RG also increased in the RG group. This may indicate that familiarization with any product can increase liking, which could be counterproductive to WG consumer acceptance if a consumer is exposed to WG and RG simultaneously. Additionally, familiarization with product taste and education about benefits in WG white whole wheat flour products, brewers spent grain products, and WG pasta aided in consumer acceptance (60, 62, 64).

*d.*    *Preference*

In a randomized control trial, De Leon et al. (73) found that initial preference for WG or RG foods did not predict consumption of WG. However, after exposure to WG foods over 6 weeks, those initially preferring RG experienced an increased positive implicit association with WG. College and university students shared that they choose WG over RG bread when given the choice (60, 70), and college students who were habitual WG consumers preferred the darker color and presence of visible grains in WG bread to RG bread (60). Additionally, those college students who reported being WG consumers gave higher liking for WG foods (70). This may indicate that familiarity with WG aids in preference. When given the option of a mixed-sorghum dish and a mixed-rice dish, Hicks-Roof et al. (61) found that University students had a similar preference for both dishes while not indicating whether any of the students had previous WG familiarity. Jahns et al. (67) found that preference for and against WG were both facilitators and barriers to WG consumption in fifth-grade Native American children.

Akaichi et al. (76) choice experiment found that appearance alone could influence preference for a hybrid rice, and Johnson et al. (65) found that consumer preference for color, nutrition, and local all influenced WTP for a high-antioxidant sweet corn. Similarly, Drugova et al. (74) found that preferences for organic bread and cookies increased with increased inclination for organics, including the beliefs that these products were superior to conventional and were not too expensive. They also found a significantly higher concentration of respondents who noted that they or someone in their family had wheat/gluten intolerance or celiac disease in the “very likely” category for organic bread purchasing. The authors note that this may suggest that wheat and gluten avoidant consumers may be substituting organic wheat products for conventional products.

*e.*    *Attitude*

Low-income adults reported a positive attitude toward the taste and texture of WG foods in a mixed methods study design (69), and two additional studies found that a more positive attitude toward certain grain products, such as believing that including a variety of WG in the diet is important, resulted in greater consumption and prioritization of those products including WG in college students (70) and organic bakery products in consumers with a higher inclination for organics (74). Interviews conducted by Leak et al. (78) after an intervention in which participants received WG foods for 16-weeks, found an improved positive attitude toward WG as a result of the familiarization. In their randomized controlled trial, De Leon et al. (72) also showed that in the group receiving WG foods for 6-weeks, willingness to include WG in their diets improved after a 6-week exposure to WG foods, but willingness to include RG in the diets also improved in the group receiving RG foods for 6-weeks. In a similar randomized controlled trial (73), found that 6-week exposure to WG foods resulted in improved implicit attitudes toward WG foods in those initially preferring RG foods. This may indicate that segments of the population display certain inherent attitudes toward WG, but that these attitudes are malleable.

Wongprawmas et al. (62) survey identified two segments of university consumers when it comes to attitudes toward WG pasta, including the “Uninvolved” consumers who are less interested in WG pasta attributes, and the “Health-conscious” consumers who have a more positive attitude toward WG pasta including believing that WG pasta is more beneficial, essential, easy to prepare, tasty, and filling. When it came to their willingness to change and include more WG pasta, the “Uninvolved” consumers were motivated by affective attitudes such as the pleasure of eating WG, while both the “Uninvolved” and “Health-conscious” consumers were motivated by cognitive attitudes such as WG health and nutritional benefits. Akaichi et al. (80) experimental auction found that specific attitudes on the environment, price sensitivity, and food origin all impacted WTP for a hybrid rice with various attribute labels.

1. *Sociodemographic characteristics*

Consumer sociodemographic characteristics were less important as factors influencing consumer acceptance of grains in general. Several studies noted the influence of sociodemographic characteristics in the context of WGs, such as food security status, which we are using as a proxy for income, as well as country of origin and household size. We report on these factors below.

*a.*    *Income/Food Insecurity*

Results from several epidemiological studies indicate that low food secure adults purchase and consume less WG products and more RG products via 2005–2012 NHANES and 2018 Nielsen Homescan data (58, 81), and only high-income adolescents experienced the modest trend in increased WG consumption (0.25 oz. eq/day) between 2005 and 2011 as compared to low-income adolescents, based on 2005–2012 NHANES data (82). The authors speculate that WG cost may be a barrier for lower socioeconomic groups, in addition to other factors. Two mixed-methods studies indicated that low-income adults have difficulty in identifying WG foods and understanding DGA guidelines (68, 69), indicating that intake and WG identification may be both associated with income and food insecurity. In their choice experiment, Dominick et al. (63) found that the number of respondents who were food secure and chose ready-to-eat-breakfast cereal because it is inexpensive was larger than those who are food insecure and chose ready-to-eat-breakfast cereal because it is inexpensive. While this study did not indicate whether the cereal was WG or RG, we are unable to synthesize this finding with speculations on income/food insecurity.

*b.*    *Household size and country of origin*

Five studies examined how ethnicity and household size, described as households with or without children, impacted consumer acceptance of WG. In an epidemiological study looking at Nielsen Homescan 2018 data for WG and RG purchases in packaged food purchasing, Dunford et al. (81) saw that households with children purchased a slightly higher proportion of products containing RG compared to WG. Dominick et al. (63) found that the number of households with children who chose ready-to-eat-breakfast cereal because “it is easy” was larger than the number of households without children, but overall, they found that of the households that reported family members eating ready-to-eat-breakfast cereal, 48% reported adults only, 32% selected adults and children, and 2% said children only as the cereal consumers. However, as Dominick et al. (63) did not indicate whether the cereal was RG or WG, we are unable to synthesize any conclusions between these studies.

Dunford et al. (81) saw small but significant ethnic differences in consumption of WG as compared to RG products, with Hispanic households purchasing more WG products than non-Hispanic households, and non-Hispanic Black households purchasing a lower proportion of RG products to non-Hispanic white households. Similarly, Hicks-Roof et al. (61) found that Hispanic participants had a higher odds of preferring sorghum to rice over white, Black, and other races. In a study utilizing interviews of shoppers at small and medium convenience stores, Lenk et al. (83) found that a subset of consumers (non-Hispanic Black, BMI = 30 kg^2^/m) was more likely to purchase less healthy products from small and non-traditional stores including sweet baked goods. Lin et al. (59) saw a higher valuation of the novel-carbon label on bread in liberal, non-White, educated consumers in their choice experiment examining these factors. These studies indicate heterogeneity when it comes to the impact of country of origin on WG consumer acceptance.

*c.*    *Age*

While age was examined less as a factor impacting consumer acceptance of WG, a handful of studies mention its impact on WTP and WG knowledge. Older participants were less WTP a higher premium for a low GHG emissions, food miles and local hybrid rice compared to a conventional rice with none of these attributes (76), similarly to Johnson et al. (65) who found that younger consumers had a higher WTP for a high-antioxidant sweet corn. Conversely, Combest and Warren (60) found through focus groups that older, non-traditional college students with higher incomes were more willing to pay for more nutritious products when asked about brewer’s spent grain products compared to younger college students. Age was not associated with the ability to identify WG correctly in low-income adults (69).

*d.*    *Gender*

Gender was also mentioned less in regard to its impact on consumer acceptance of WG. Akaichi et al. (76) found that female participants were WTP a lower premium for a local hybrid rice compared to a non-local conventional rice compared to male participants. De Leon et al. (72) found differences between male and female participants in how they rated WG in terms of appearance, flavor, texture, familiarity, perceived healthiness, and willingness to include WG in their diet. On the other hand, Lenk et al. (83) found that country of origin and education level influenced purchasing of unhealthy foods including less WG, but they did not see a significant difference between male and female shoppers.

*e.*    *Education level*

Four studies mentioned education level as a factor impacting consumer acceptance of WG. Participants with more education were WTP a higher price premium for both hybrid rice and a novel-carbon labeled bread compared to less educated participants (59, 76). Education level in low-income adults did not impact their ability to correctly identify WG foods, but it approached significance (*p* = 0.008) (69), and Lenk et al. (83) found that interviewees with less education had higher purchases of unhealthy foods at convenience stores including less WG. As a result, the impact of education on consumer acceptance of WG displayed heterogeneity when it comes to impact on WTP, knowledge, and type of food purchase.

1. *Environmental factors: food outlet, home, and community programs*

The fourth category, environmental factors, describes how food outlets, home settings, and community programs all impact consumer acceptance of WG with several studies examining these factors. While there are various places consumers buy grains, fewer WG products are purchased by adults and adolescents at small, non-traditional, and convenience stores and cafeterias (83), whereas Setiono et al. (57) showed that more WG are purchased at grocery stores. However, this NHANES analysis (57) inferred this based on their conclusion that grain product purchases at convenience stores had less fiber and more carbohydrates, and therefore they surmised that these purchases were less likely to be WG. Johnson et al. (65) found that WTP for a high-antioxidant corn variety was highest at health food stores and wholesale club stores, indicating a better environment for acceptance of this item. Two studies found that lack of availability of WG at home was a significant barrier to WG consumption (66, 67). As a result, food outlet type and availability seem to impact WG consumer acceptance including WG presence in the home.

One study examined how community programs influence WG consumer acceptance and found that overall grain purchasing marginally decreased in four families during their participation in a 16-week fresh fruit and vegetable CSA community supported agriculture program in Utah (84). They speculate that grains were replaced by vegetables and fruits in the diet during the program months. As these CSA programs likely do not aim to decrease grain consumption, knowing this impact could aid programs in simultaneously supporting grains.

1. *Interventions to increase consumer grain acceptance*

While the majority of the included studies have examined the factors impacting WG and other grain consumer acceptance, a handful of the studies also conducted interventions to examine these factors (*n* = 10) (Table 3). We define interventions as any activity or treatment given to participants to examine alterations in consumer acceptance of grains (31% of included studies are linked to interventions). We categorized the interventions into the following categories based on the type of activities they employed with some studies falling into more than one category: exposure and familiarization, educational programing, and information provision.

Four intervention studies were categorized as exposure and familiarization and showed that exposure to WGs and accompanying information led to increased consumption and liking of WG in adults (72) and children with prediabetes and obesity (78), improved positive implicit association with WG taste in adults initially preferring RG (73), and increased acceptability and similar sensory scores ratings of WG white whole wheat flour products to identical all-purpose flour products in college students (64). De Leon et al. (72) randomized control trial in which one group received WG for 6-weeks and another group received RG for 6-weeks, found that sensory acceptance, liking, and attitudes improved for both groups. Leak et al. (78) conducted interviews with caregivers of youth diagnosed with obesity and prediabetes who had received 16-weeks of WG as part of a FoodRx program as well as WG information on their benefits and preparation (optional cooking classes, recipes, and instructional videos), and found that the program resulted in increased knowledge of WG benefits, more positive attitudes toward WG, better WG purchasing behavior, and more resources for WG preparation and ways to incorporate WG in meals for children. De Leon et al. (73) conducted a 6-week pilot randomized controlled trial with one group receiving WG products and the other group receiving RG products and found that exposure to WG foods led to improved automatic attitudes. Samuel et al. (64) found that sensory acceptance of white whole wheat flour (WWWF) baked goods improved in college students after familiarization with health and sensory profiles of WWWF products and did not differ from the all-purpose flour analogs.

Three interventions which also fell into the exposure and familiarization category found that providing taste-testing or taste assurances resulted in increased acceptability of WG products (77, 79) and organic specialty bakery products (75) as a result of familiarization with these products. Yao et al. (77) conducted a quasi-experimental 4-week trial in which one group of food pantry clients received oral messaging about WG intake recommendations, tasted a chicken-and-whole-grain pasta dish and were then invited to make the recipe at home with ingredient and recipe provision and one group acted as the control. They found in follow up interviews that the group who tasted the dish and were given resources had improved perceptions of WG intake and self-efficacy when it came to preparing WG as compared to the control. MacNab et al. (79) conducted a nutrition education intervention with community residing adults (aged 60 years and older) involving WG taste-testing as well as educational programing and found that average knowledge and intended WG intake increased in participants. Drugova and Curtis (75) conducted a choice experiment to understand consumer WTP for organic wheat bakery products (bread, cookies, croissants) and found that providing taste assurances on these products resulted in an improved WTP for these organic wheat bakery products in certain consumer segments.

Seven studies examined the effect of WG information provision on WG consumer acceptance, including Yao et al.’s intervention at a community food pantry involving verbal WG messaging about WG recommendations and recipe provision (77), Arts et al. (85) intervention utilizing educational WG text messages and point-of-sale cafeteria messaging to college students about the benefits of WG and reminders to consume them, information about the health benefits and expected sensory profile of WWWF pastries (64), and information accompanying bi-weekly deliveries of WG for 16 weeks including optional cooking classes, calls, recipes, and instructional videos (78). All four of these interventions involving information provision saw increased WG intake and acceptance. However, in two separate studies utilizing information provision, neither a summary of nutritional benefits for ready-to-eat-breakfast-cereal from a choice experiment by Dominick et al. (63) nor Drugova and Curtis (75) provision of education on organic labeling standards for organic specialty bakery products altered WTP for these grain products (63, 75). This is compared to one experimental auction which found that exposure to information via labeling on greenhouse gas emissions, food miles, and origin of food resulted in differing WTP amongst consumers for a hybrid rice (76). As a result, the type of information provision might change aspects of consumer acceptance (such as attitude or intake) of grains while other aspects (such as WTP) may remain unchanged. Additionally, while Leak et al. (78) indicated that while WG exposure and familiarization as well as messaging provision was beneficial, barriers to WG consumer acceptance still remained after the intervention including poor experiences cooking WG due to their novelty, and lack of resources to prepare novel whole grains even with educational videos and recipes.

One intervention was categorized as educational programing, in addition to exposure and familiarization as reported above, in which MacNab et al. (79) provided theory-based educational lessons and hands-on activities to U.S. community residing older adults through three, 1-h sessions, including participant-instructor interaction, small group discussion, hands-on-activities, and taste testing. They found that mean WG knowledge score increased as well as mean WG liking and intended intake from pre- to post intervention but noted that these results were limited by potential recall bias and self-reporting error.

### Consumer engagement with alternative grain networks

We will now report the findings that address our secondary research questions starting with RQ2b [How do U.S. consumers engage with grains including through the commodity grain system or alternative grain networks (AGNs)]. Three qualitative case studies by Baker and Russell (86), Mars (87), and Meyer et al. (88) utilized a case study model to document consumer engagement with AGNs in New York, Arizona, and Colorado respectively (RQ2b). However, there were no papers which reported on similar aspects in industrial or commodity grain systems or even utilized this terminology. Baker and Russell (86) conducted a case study of the Northeast regional grain system, including an articulation of the coordination required amongst grain value chain members for successful partnerships, infrastructure establishment, and local and organic grain market creation, through documentation of a New York farmer, miller and farmer partnership, and community supported bakery (CSB), a type of CSA bread subscription program. They qualitatively document the growth of consumer demand for local and organic small grain markets since the early 2000s, consumers’ interactions with the CSB, how these AGNs make local grains accessible to consumers, and how shoppers at the New York State and GrowNYC’s Greenmarket were able to engage with AGNs due to the local policy that at least 15% of the grains used in baked goods sold at the market be locally sourced, which was later raised to 25% in 2019 (86, 89). As part of his case study, Mars (87) conducted interviews and participant observations to understand how a Southern Arizona community supported baker utilized entrepreneurial leadership to embed the bakery’s consumers in the AGN, empowering them to be co-creators of local food systems, marketers of his breads, and promoters of local grains. While documenting entrepreneurial leadership is outside of the scope of this paper, its values to aid local food entrepreneurs in increasing their income while strengthening their local food system is relevant, as Mars documents that the CSB was able to successfully embed his consumers into co-creation of both the bakery as well as the local food system through establishing baker-customer relationships, facilitating ownership through consumers helping set-up the weekly bread pick-ups, and establishing community amongst consumers through regular interaction at pick-ups. Meyer et al. (88) conducted a multiple-case study analysis of AGNs in South Tyrol, Italy, and Colorado, to qualitatively document various factors including geographical proximity, cultural homogeneity, commercial viability, and community involvement. While the findings related to Italy are outside of the scope of this review, the Colorado findings had relevant findings and utilized semi-structured interviews with AGN stakeholders, consumers, and key informants who founded the AGN. Some key findings across all three studies include that general consumer awareness of AGNs is limited, and that consumers report purchasing through the AGN to support small business and communities, relationships, and due to search for alternative products.

While not citing quantitative numbers, all three case studies document that consumer interest in AGNs has been growing since the early 2000s in the Northeastern U.S., Arizona, and Colorado. All three studies noted that coordination along the value chain between producers, millers, distillers, brewers, and bakers was essential to deliver high quality local grain products to consumers. Additionally, all three articles noted that sustained consumer engagement and ownership of AGNs was key for their success, with Meyers et al. (88) documenting this through the Colorado AGN membership and Baker and Russell (86) and Mars (87) documenting how the subscription-type bread service of two CSBs embedded consumers in the co-creation of AGNs.

### Consumer acceptance of local grains

We next document findings on RQ2a: What are the key factors influencing U.S. consumers’ acceptance of local/regional grain products? We note here that while the original research question included “local/regional grains” language, we will simplify to the language of “local grains” for clarity going forward.

The same three articles that examined consumer engagement with AGNs documented aspects of consumer acceptance of local grain products including local bread, flour, and bulk grains (86–88). All three articles noted that consumers of these AGNs preferred locally sourced grains and bread. Baker and Russell (86) and Mars (87) document how the CSB aids in increasing support of local grains through giving the consumer ownership as a co-creator of their local grain network. All three case studies qualitatively document that consumer demand for local small grains and grain products such as alcoholic beverages, bread, baked goods, and pasta has been growing since the early 2000s. Baker and Russell (86) indicated that more research was needed to determine whether market demand for local grain products would continue to grow, and Meyer et al. (84) noted that a growing amount of consumers were interested in traditional bread products.

While some consumers are WTP a premium for local bread (86, 88), Mars (87) noted that based on consumer feedback, the CSB had reached price point saturation at the time of the case study, limiting the ability to include additional, more expensive local flour in their products. Both Baker and Russell (86) and Mars (87) only noted qualitative observations on WTP, while Meyer et al. (88) offered quantitative ranges as a result of semi-structured interviews with consumers. In addition, Baker and Russell (86) note that local grain market saturation and continued consumer interest in paying premiums for local grains is unclear. Meyer et al. (88) and Mars (87) both noted that consumers preferred the taste of local bread, with Meyer et al. (88) noting additional desirable product attributes such as nutrition, artisanship, authenticity, and WG content.

## Discussion

We discuss the key findings of our scoping review, including major themes regarding consumer acceptance of grain foods as well as documentation of consumer engagement with AGNs and the local grain products they produce. These insights inform our recommendations for future studies and policy, industry, and academic engagement.

### Key factors influencing U.S. consumers’ acceptance of whole grains

In addressing the first research question (RQ1: What are the key factors influencing U.S. consumers’ acceptance of grains including reasons that motivate or deter consumption), we found that the key factors impacting U.S. consumer’s acceptance of WGs included taste, price, knowledge, familiarity, attitude, and income/food insecurity, as well as interest in specialty categories such as organic. As many of the barriers identified were those indicated in prior country-specific studies (42–45) and one global narrative review (48), either previous interventions have been ineffective at achieving desired aims, or interventions have largely not been implemented, indicating a need for action. Additionally, the barriers and drivers to WG consumption between the U.S. and other countries are largely similar, indicating that we can broadly utilize recommendations from these other studies to leverage interventions toward increasing U.S. consumption. However, it is important to note that low and middle income countries (LMIC) countries face additional barriers including convenience, because while many prepared WG products exist in the U.S. (91), cooking raw grains is often the most common option in LMIC. This scoping review reports on general descriptive trends of consumer acceptance factors seen in the included articles, as it is beyond its scope to provide linear, directional recommendations or insight on how factors interact dynamically due to the paucity of articles on each factor.

Taste as a key barrier to WG consumer acceptance is supported by the literature (92–95), and while taste perceptions can vary by population (45), familiarization/exposure, taste assurances, grain breeding for whole-grain end-use, taste-testing, and intentional product formulation can change taste perceptions and consumption patterns (25, 40, 46, 72, 73, 95). Cost was also indicated as a barrier to WG consumption in previous studies (42, 96), and some suggest that policy changes to reduce cost could aid in increased WG consumption, especially in lower-income populations through public aid programs (48). Organic is another attribute of interest, and while organic grain products are not necessarily WG, it is an important attribute to note as organic products have seen record high sales growth of $71.6 billion in 2024, a 5.2% increase from the previous year (97). Specifically related to grains, “bakery and fresh breads” was the largest category within grocery, and grew by 2.2%, driven by interest in sourdough. Due to organic wheat flour being an expensive input, it is beneficial for practitioners to know that to see increased sales in consumers not already committed to organic, taste assurances are the most effective intervention compared to organic education (75).

Sociodemographic factors and food outlet availability were additional factors impacting consumer acceptance of WG through accessibility and availability of these products. While our included studies mentioned the impacts of various sociodemographic factors, income/food insecurity, country of origin, and education level were mentioned the most often with heterogeneity across these factors and their impact on consumer acceptance of WG. Food insecurity and income level are known to impact healthy eating across food groups, and policy change and advocacy to make WG more accessible is key to increasing their consumer acceptance (68, 69). Food outlet availability is another factor that influences WG purchasing and consumer acceptance. While only mentioned by two studies (57, 58) in our scoping review, food environment equities influence the availability and accessibility of WG. Similarly, availability of WG products at home is a barrier to intake with a study by Rosen et al. (98) showing that WG availability at home was a bigger barrier to intake than psychosocial factors in elementary children. Additionally, studies show that there is elevated prevalence of obesity in rural children, and tribal reservations have less access to health food such as WG products, emphasizing the need for food environment interventions that make healthy food accessible and affordable (67, 99, 100). In addition, only one included paper (71) mentioned WG consumption in schools, with other reviews calling for these as primary outlets for getting WG to children (48).

In addition to the impact of sociodemographic factors and food outlet availability on WG consumer acceptance (42, 48), the availability of WG products on the market has also been questioned regarding consumer acceptance, although this was not examined in our included studies. Some sources offer that WG product availability has increased, such as Sluyter et al. (90), who analyzed the Oldways Whole Grains Council (WGC) Stamped Product database and found that between 2008 and 2020, products using the WGC stamp increased their mean WG amount by 36%. This is seen in the fact that while there were 50 products using the stamp in 2005, this grew to more than 10,700 products in 2020. Conversely, Metcalfe (25) noted that as the commodity grain system in the U.S. is set-up to make RG products from the breeder to the miller and baker, 95% of wheat is sifted in the U.S., resulting in the majority of grain products to be in their RG form. However, cereal breeding labs like the Washington State University Breadlab in Washington specifically breed grain with WG as the end-use, thereby breeding for optimal nutrition, flavor profiles, and the ability to taste terroir, or a sense of place, in the grain (101). Additionally, not addressed in our studies was the topic of WG and ultra-processed foods (UPFs). Many WG products and breads would fall into the UPF category per NOVA classification, posing a potential public health crisis regarding consumer perception and intake of these WG products, resulting in negative health outcomes. However, studies such as Price et al. (102) and Price et al. (103) found that high-WG foods considered as UPFs via the NOVA classification system are not associated with the deleterious cardiometabolic risk factors typically accompanying UPFs. As a result, guidance on how to discourage UPF consumption while still encouraging WG consumption is needed, such as better UPF classification schemes that don’t erroneously vilify healthful foods such as WG and this should be taken into consideration before endorsement of current schemes.

In harnessing the factors most impacting consumer acceptance of WG to increase engagement, although our included studies did not explicitly note the impact of public, private partnerships (PPP) (relationships between government, industry, academic and non-profit groups) on increasing consumer acceptance of WG, studies in other countries found that PPP have been the most successful at increasing WG intake (49, 104). This is demonstrated by both the Danish Fuldkorn program (105), which successfully increased the average Danish citizen’s WG intake to an average of 82 g/day, and the new Swedish FullkornsFrämjandet WG program (106). PPPs as effective organizational approaches to increasing WG consumer acceptance was also recently endorsed by the international Whole Grain Initiative 2025 Declaration of Acts report (107). As a result, PPPs that could harness our identified factors of taste, price, knowledge, familiarity, attitude, and income/food insecurity could be effective in increasing WG consumer acceptance. In addition, based on our findings, multi-stakeholder campaigns that could help with consumer identification of WGs, including the definition of a WG, what counts as a WG food, WG benefits, and how to use labeling to discern the WG content of a cereal would be beneficial. We recommend feasibility studies to examine the most actionable methods as this is outside the scope of a scoping review (108). Upon analysis of 2003–2018 NHANES data, Du et al. (109) found that the differing WG food definitions from leading U.S. institutions resulted in disparate outputs for U.S. estimated intake, determination of WG foods, and associated WG food consumption trends. Furthermore, WG labels currently utilized by industry are not regulated by the FDA, which can lead to consumer confusion. As a result, a standardized WG definition and labeling requirement across U.S. regulatory, industry, and governmental groups is needed which would aid in establishing consistent associated trends in WG consumption.

### Key factors influencing U.S. consumers’ acceptance of alternative grain networks

In addressing our two secondary research questions regarding consumer acceptance of local grain products and engagement with AGNs, there were only a handful of studies reporting on these topics. These findings offer a layered understanding of each specific AGN system through documenting observations, interviews, and quantitative findings to provide insights into the burgeoning world of local grain consumer interest. Regarding RQ2b (How do U.S. consumers engage with grains including through the commodity grain system or alternative grain networks (AGNs)?), it is important to note that while there were no articles utilizing the language of consumer engagement with the commodity grain system, likely due to syntax and a lack of its usage in consumer affairs, most of the included articles documented consumer engagement with the commodity grain system. However, three of our review’s articles qualitatively documented consumer engagement with AGNs (86–88) utilizing a case study format which poses certain strengths as well as weaknesses.

Although there is a paucity of academic literature documenting AGNs in the U.S., thriving networks are represented by organizations such as the Artisan Grain Collaborative, Common Grain Alliance, WSU Breadlab, Glynwood Center for Regional Food and Farming, Maine Grain Alliance, and the Colorado Grain Chain, with some grey literature to support them. Additionally, articles documenting other aspects of AGNs besides consumer acceptance exist both in the U.S., and globally (110–117). These articles and grain networks describe AGNs as grain systems which create local markets for value-added, regionally grown small grains, re-regionalizing grain production, processing, and eating onto the landscape through diversifying farmer rotations, establishing localized intermediate processing such as grain cleaning, milling, and malting infrastructure for grain food and beverage end-use, and embedding local bakeries, retailers, breweries, and distilleries into communities (13, 114). AGNs may also focus on sustainable agricultural practices, value seed and food sovereignty, connect consumers to their food and those who made it, strengthen enterprise and farm-level resilience, diversify farmer income through value added products, and coordinate relationships amongst grain chain actors for enhanced product flows (114, 118–120). In addition, they can re-value farmer- and rural-ways of knowing and being.

While documented in a qualitative manner in the included case studies, all three noted consumer interest in AGNs and their grain products, while none provided a quantitative assessment of the potential local grains market growth (86–88). Stedden et al. (114) document that while there is increased interest in more mainstream organic grains and oilseeds (soybeans and corn), with the U.S. supply unable to currently meet that demand, demand for wheat, oat, rye, and barley is also growing due to increased interest in local food systems (13). Kissing Keucek et al. (121) also document a burgeoning interest in local grains and the AGNs which produce and prepare them in the U.S., with additional literature pointing to their demand in Europe and the United Kingdom (110, 111, 122–124). Along with the broader literature on AFNs, all three studies noted that consumer engagement with AGNs as equally important stakeholders is crucial for their sustained development and continued demand, as grassroots and alternative governance structure movements can only continue so far as there is continued interest and investment from all members including consumers as their co-creators (125). However, the burden of advancing AGNs cannot fall solely on the shoulders of consumers, as they are only one member of the grain value chain and are already faced with countless food product purchasing decisions. As a result, for increased consumer engagement with AGNs, we recommend strategies that not only directly raise consumer awareness through education on the nutritional, agronomic and community benefits of AGNs, and embodied taste testing and sampling experiences to increase familiarization with the products they offer as demonstrated and called for by others (126, 127), but also strategies that target activity across the rest of the value change including supporting infrastructure needs and grain market hubs (128), and utilizing policy change and research to better understand needs (129).

Areas for future research and policy action to increase consumer engagement with AGNs include policy reviews and impact, economic, and market research to understand how AGNs have been affected and made an impact in these three categories so as to make informed decisions about future actions. In addition to these efforts, quantitative documentation of U.S. AGNs in the academic literature would be beneficial for a greater mapping of these networks and legitimizing of them in academic circles, redirecting funding toward supporting AGN research and outreach, which historically have been under-supported alongside other AFNs (130), and conducting market research to assess market growth for these AGNs, similar to what Platel and Russell (14) did for New York State in 2015 (97), would all be beneficial for advancing these networks. Policy review studies that assess the impact of regional grain policies such as those in the Northeast and other regions would be beneficial to better understand these policies’ effectiveness. Examples include assessing the economic and market impact of policy efforts such as the GrowNYC Greenmarket rule that requires bakery products to contain 25% local grain (89), state policy tax incentives such as the 2014 NY Craft Act (131), and policies the support the development of AGN infrastructure such as the USDA Farm Storage Facility Loans (132) and Resilient Food System Infrastructure (133) technical assistance programs^1^ to establish the effect that these policies have had on establishing AGN infrastructure, creating demand and markets for local grains, and benefiting farmers, processors, end-users and consumers in each region. Understanding this impact will allow for future policy recommendations, such as continued tax incentives for business incorporating a minimum amount of local grains, and on a broader level, reform to policies surrounding crop subsidies. Economic impact studies such as the ‘Report on Economic Impact of Craft Brewing on the State of Vermont’ (134) are needed for AGNs to better understand the economic impact of regional grain networks both to use this information as an advocacy tool as well as to better target future efforts.

### Key factors influencing U.S. consumers’ acceptance of local grains

The main factors consistent across all three articles for consumer acceptance of local grain products (RQ2a: What are the key factors influencing U.S. consumers’ acceptance of local/regional grain products?) were loyalty to AGNs, value for community and supporting small businesses, accessibility to local grains as a result of AGN value chain partnerships, and taste for local bread. The included studies displayed heterogeneity in the premium that consumers are WTP for local bread, and commented on the need for equitable access but did not mention available programing. However, AGNs are working to find innovative ways to increase equitable access to these products through programs such as “Neighbor Loaves,” which started during the COVID-19 pandemic and continues into the present at various bakeries across the country, providing customers with opportunities to purchase regionally sourced loaves of bread for food pantries (135) Additional mechanisms for equitable access include some farmer’s markets programs which allow SNAP EBT users to use SNAP benefits on almost all food items at a farmer’s market (excluding hot, prepared items, and soap and flowers), increasing consumer access to local grains (136). Supporting the continuation and expansion of this program to more markets, along with the SNAP Double Up program, which is available in 25 states and doubles the amount that SNAP users can spend at a market, can also help with equitable access to local grains (137). Finally, AGN leaders call for a deeper dive into the SNAP funding structures and criteria that must be met for a local business to offer SNAP benefit redemption for their goods, potentially offering an additional avenue for increased equitable access to local grains (personal correspondence, 2025).

Similar to the organic wheat product category, our studies showed that there is a select segment of consumers who are interested in local grain products, but they did not go into detail on these consumer profiles However, a recent consumer survey of 1,500 census-representative U.S. adults conducted in 2025 by the Whole Grains Council (WGC), Artisan Grain Collaborative (AGC), and University of Minnesota (UMN) found that two out of five consumers value local grains, two out of five are indifferent, and the remainder do not value local grains. They segmented consumers into three groups when it comes to how they value the environmental, social (e.g., community support), and practical (e.g., nutrition and freshness), aspects of local grains: local grain enthusiasts, local grain value skeptics, and local grain idealists (138). The survey also showed that cost, limited availability, and hard-to-identify were the main barriers for purchasing local grains, while taste opportunities, better labeling and signage, and information on true cost would all be motivators. A survey of 202 NY GrowNYC Grainstand customers found that the majority of consumers bought local grains and dry beans because they wanted to support local growers, and that increased educational opportunities, tasting experiences, and local grain availability would help them purchase more products (127). Farmers, millers, bakers, and retailers who sell direct-to-consumer can utilize this information to tailor their marketing strategies for increased consumer acceptance of local grains, as well as policy advocates who can build policy cases based on the surveys’ findings around the need for greater consumer educational and tasting experiential opportunities. Baker and Russell (86) and others (114) also note that along with direct consumer interest, the coordinated partnerships along the value chain are what allow consumers to access organic and local products made with high quality grains and flours, emphasizing the importance of involvement and action by all grain value chain stakeholders for increased consumer acceptance of these local grain products.

As follows, policy action and research are key for advancing consumer acceptance of and access to local grains. Regarding future research, while nutrition was not noted as a key factor, local grain products have the potential to be delicious, nutritionally dense components of a healthy meal, as they often offer a diversity of heritage and heirloom grains to consumers, distinct from the few modern varieties of rice, wheat, andcorn that consumers commonly eat (25). In addition, these AGNs typically involve craftsmanship in their production such as stone-milling and long-fermentation which can create value-added traditional local grain products and elevate these products from their often-discounted view as ubiquitous and standardized, to a featured spot on the menu. As follows, AGN leaders note that studies examining the nutrient density of local grains could aid mills, bakers, and retailers in labeling their products with accurate nutritional information as well as potentially give them a marketing edge if the local flour and grain products show greater nutrient density (personal correspondence, 2025. Many factors would need to be considered in such a study including growing conditions, varietal type, region, and product processing. As no included studies reported on U.S. consumer interest in alternative grains such as heritage wheats or diverse grains such as oats or barley, or pseudocereals such as quinoa or amaranth, academic research examining this relationship could aid in better understanding consumer markets. This research could be modeled off studies in Canada (139) and Europe (140, 141) which document such interactions, as well as a study in the U.S. which was outside of our inclusion criteria as it utilized trained panelists (121). Additional studies include a systematic review on consumer acceptance of these grains globally (142), as well as consumer insight surveys which did not meet our inclusion criteria, such as the WGC survey (143) and the 2023 Ardent Mills survey examining consumer interest in ancient grains (144). All of these resources document a segment of consumers who are interested in these products. Additionally, although local grain products are not inherently WG, a study examining how often local grains are offered in their WG form could aid in better understanding how local grain products contribute to health and substantiate the evidence for their purchase and inclusion in local food systems.

### Limitations

There are limitations inherent to scoping reviews. We do not rank the quality of the evidence for the included studies, because this is not methodologically appropriate for a scoping review (53, 108). Clinical decisions, therefore, should not be based on this and other scoping reviews alone. As it is also outside a scoping review to provide a feasibility assessment, we encourage layering these findings with feasibility assessments of included recommendations for most actionable results. There is also the potential for publication bias in scoping reviews as studies with negative results are reported less often, resulting in a scoping review that broadly maps the landscape with only positive and statistically significant findings. In addition, since we excluded gray literature, publication bias is potentially greater.

Consumer perceptions are subjective and should be taken accordingly. There were most often only a few studies reporting on each attribute (other than the WG category) so generalizing the findings should be conducted with this in mind. Additionally, the study designs displayed a great deal of heterogeneity, ranging from focus groups to randomized control trials to observational studies. As a result, the findings are not directly interchangeable, unlike the findings in a meta-analysis. However, the descriptive findings are still valuable for mapping the current evidence base and making research recommendations. Additionally, the three articles examining consumer acceptance of AGNs and local grains were qualitative case studies, which could provide more of a subjective view of the analysis and findings.

Our exclusion criteria eliminated food science and technology articles looking at trained sensory evaluation and formulation of grain foods in a laboratory setting, potentially excluding articles which could have contributed sensory acceptance findings on grain products. However, as we wanted to examine untrained consumer acceptance of grain products already on the market in a non-laboratory setting, these food technology and trained food sensory evaluation studies in a laboratory setting are outside of the scope of our study. However, we recommend a follow up scoping review mapping the WG and grain product food formulation and food science specific research landscape. Finally, our search was limited to English language publications which may have limited our findings. However, as our review was focused on U.S. consumer acceptance, there is likely less impact from this limitation as opposed to a review focusing on global consumer acceptance.

## Conclusion

The current challenges facing food systems call for a transition from our current model to one that supports both planetary and human health. Grains, a historically grown and globally consumed staple crop, remain as a core component of human diets. Increasing the diversity of grains grown in regionalized food systems, as well as in the human diet in their whole grain form can aid in a transitioned and enhanced food system. This scoping review documents U.S. consumer acceptance of grain foods and engagement with alternative grain networks and resulted in understanding the key factors impacting consumer acceptance of whole grains, local grains, and alternative grain networks. The key factors mentioned most frequently in our included articles for whole grain consumer acceptance included taste, price, knowledge, familiarity, attitude, and income/food insecurity, and interest in specialty categories such as organic. Future research and policy initiatives must focus on these factors as key levers for increasing whole grain consumption in the U.S. Additionally, this review found that consumer acceptance for local grain products through engagement with alternative grain networks was based on loyalty to alternative grain networks, value for community and supporting small businesses, and taste for local bread. These alternative grain networks offer an additional way to enhance the grain-based system and transition our food system to one that is equitable, just, nutritious, and ensures planetary flourishing now and into the future. We make policy and research recommendations for the advancement of these alternative grain networks and the local grains they produce, including policy and economic impact reviews to understand how local grain policies have impacted the landscape to date, future research to explore nutrient density of local grains as well as methods for increased equitable access, and continued support for grain value chain infrastructure programs.

## Data Availability

The original contributions presented in this study are included in this article/Supplementary material, further inquiries can be directed to the corresponding author.

## Footnotes

1 Other federal funding streams including SARE, USDA LFPP, USDA OMDG, USDA OREI, and other programs that are available from general regional food system and agriculture research have in cases been instrumental in supporting the development of AGNs across the country.

## References

[1] Swinburn B Kraak V Allender S Atkins V Baker P Bogard J The Global Syndemic of Obesity, Undernutrition, and Climate Change: the *Lancet* Commission report. *Lancet.* 2019;393:791-846. 10.1016/S0140-6736(18)32822-8 30700377

[2] Rodríguez-Huerta E Bell B Battaglia K Decker Sparks J Benoit Norris C Marquez A Current and recommended diets in the USA have embedded forced labour risk. *Nat Food.* 2025;6:1042-1053. 10.1038/s43016-025-01242-8 41062630

[3] Rockström J Thilsted S Willett W Gordon L Herrero M Hicks C The EAT-*Lancet* Commission on healthy, sustainable, and just food systems. *Lancet.* 2025;406:1625-1700. 10.1016/S0140-6736(25)01201-2 41046857

[4] Milani P Torres-Aguilar P Hamaker B Manary M Abushamma S Laar A The whole grain manifesto: From Green Revolution to Grain Evolution. *Global Food Security.* 2022;34:100649. 10.1016/j.gfs.2022.100649

[5] Ross A van der Kamp J King R Lê K Mejborn H Seal C Perspective: a Definition for Whole-Grain Food Products-Recommendations from the Healthgrain Forum. *Adv Nutr.* 2017;8:525-531. 10.3945/an.116.014001 28710140 PMC5502867

[6] USDA. Feed Grains Database - Feed Grains Custom Query. 2025. Washington, DC: USDA

[7] Howard PH. AFHVS 2016 presidential address: Decoding diversity in the food system: wheat and bread in North America. *Agric Human Values.* 2016;33:953-960. 10.1007/s10460-016-9727-y

[8] Jones S Econopouly B. Breeding away from all purpose. *Agroecol Sustainable Food Syst.* 2018;42:712-721. 10.1080/21683565.2018.1426672

[9] Bowles T Mooshammer M Socolar Y Calderón F Cavigelli M Culman S Long-Term Evidence Shows that Crop-Rotation Diversification Increases Agricultural Resilience to Adverse Growing Conditions in North America. *One Earth.* 2020;2:284-293. 10.1016/j.oneear.2020.02.007

[10] Davis A Hill J Chase C Johanns A Liebman M. Increasing cropping system diversity balances productivity, profitability and environmental health. *PLoS One.* 2012;7:e47149. 10.1371/journal.pone.0047149 23071739 PMC3468434

[11] Gaudin A Janovicek K Deen B Hooker D. Wheat improves nitrogen use efficiency of maize and soybean-based cropping systems. *Agric Ecosyst Environ.* 2015;210:1-10. 10.1016/j.agee.2015.04.034

[12] Janovicek K Hooker D Weersink A Vyn R Deen B. Corn and soybean yields and returns are greater in rotations with wheat. *Agronomy J.* 2021;113:1691-1711. 10.1002/agj2.20605

[13] Halloran A. The New Bread Basket: How the New Crop of Grain Growers, Plant Breeders, Millers, Maltsters, Bakers, Brewers, and Local Food Activists Are Redefining Our Daily Loaf. 1 ed. White River Junction, VT: Chelsea Green Publishing; 2015.

[14] Platel R Russell J. Meeting the Demand for Small Grains in New York State. 2015.

[15] Sonnino R Marsden T. Beyond the divide: rethinking relationships between alternative and conventional food networks in Europe. *J Econ Geography.* 2005;6:181-199. 10.1093/jeg/lbi006

[16] Low S Adalja A Beaulieu E Key N Martinez S Melton A Trends in US local and regional food systems: A report to Congress. Washington, DC: USDA, 2015.

[17] Kim K Tsao R Yang R Cui S. Phenolic acid profiles and antioxidant activities of wheat bran extracts and the effect of hydrolysis conditions. *Food Chem.* 2006;95:466-473. 10.1016/j.foodchem.2005.01.032

[18] Liu R. Whole grain phytochemicals and health. *J Cereal Sci.* 2007;46:207-219. 10.1016/j.jcs.2007.06.010

[19] Aune D Keum N Giovannucci E Fadnes L Boffetta P Greenwood D Whole grain consumption and risk of cardiovascular disease, cancer, and all cause and cause specific mortality: systematic review and dose-response meta-analysis of prospective studies. *BMJ.* 2016;353:i2716. 10.1136/bmj.i2716 27301975 PMC4908315

[20] Chanson-Rolle A Meynier A Aubin F Lappi J Poutanen K Vinoy S Systematic Review and Meta-Analysis of Human Studies to Support a Quantitative Recommendation for Whole Grain Intake in Relation to Type 2 Diabetes. *PLoS One.* 2015;10:e0131377. 10.1371/journal.pone.0131377 26098118 PMC4476805

[21] Martínez I Lattimer J Hubach K Case J Yang J Weber C Gut microbiome composition is linked to whole grain-induced immunological improvements. *ISME J.* 2013;7:269-280. 10.1038/ismej.2012.104 23038174 PMC3554403

[22] Um C Peters B Choi H Oberstein P Beggs D Usyk M Grain, Gluten, and Dietary Fiber Intake Influence Gut Microbial Diversity: data from the Food and Microbiome Longitudinal Investigation. *Cancer Res Commun.* 2023;3:43-53. 10.1158/2767-9764.CRC-22-0154 36968219 PMC10035461

[23] Afshin A Sur P Fay K Ferrara G Salama J Mullany E Health effects of dietary risks in 195 countries, 1990–2017: a systematic analysis for the Global Burden of Disease Study 2017. *Lancet.* 2019;393:1958-1972. 10.1016/S0140-6736(19)30041-8 30954305 PMC6899507

[24] Murphy M Schmier J. Cardiovascular Healthcare Cost Savings Associated with Increased Whole Grains Consumption among Adults in the United States. *Nutrients.* 2020;12:2323. 10.3390/nu12082323 32756452 PMC7469007

[25] Metcalfe M. Wheat for Community Food Systems and Equitable Nutrient Availability. Pullman: Washington State University; 2023.

[26] Wilder RM. A Brief history of the enrichment of flour and bread. *J Am Med Assoc.* 1956;162:1539-1541. 10.1001/jama.1956.72970340006010 24544167

[27] Mozaffarian D Hao T Rimm E Willett W Hu F. Changes in diet and lifestyle and long-term weight gain in women and men. *N Engl J Med.* 2011;364:2392-2404. 10.1056/NEJMoa1014296 21696306 PMC3151731

[28] Yu D Shu X Li H Xiang Y Yang G Gao Y Dietary carbohydrates, refined grains, glycemic load, and risk of coronary heart disease in Chinese adults. *Am J Epidemiol.* 2013;178:1542-1549. 10.1093/aje/kwt178 24008907 PMC3888273

[29] Semba R. The historical evolution of thought regarding multiple micronutrient nutrition. *J Nutr.* 2012;142:143S-56S. 10.3945/jn.110.137745 22157539

[30] Gropper S Smith J Groff J. Advanced human nutrition and metabolism. Belmont: Wadsworth. 2013.

[31] Fletcher R Bell I Lambert J. Public health aspects of food fortification: a question of balance. *Proc Nutr Soc.* 2004;63:605-614. 10.1079/pns2004391 15831133

[32] Junod S. Folic Acid Fortification: Fact and Folly, Part III. FDLI Update. USDA: Washington, DC;2001:25.

[33] FDA. 21 CFR 137.165 Enriched Flour. Silver Spring, MA: FDA;1993.

[34] FoodData Central. Flour, whole wheat, unenriched. 2025. USDA: Washington, DC

[35] USDA. Nutrition and your health: Dietary Guidelines for Americans, 2005: part D: science base: section 5: carbohydrates. Washington, DC: United States Department of Agriculture 2005.

[36] USDA, HHS. 2020-2025 Dietary Guidelines for Americans (DGA). 2020. USDA: Washington, DC

[37] Cheng W Sun Y Fan M Li Y Wang L Qian H. Wheat bran, as the resource of dietary fiber: a review. *Crit Rev Food Sci Nutr.* 2022;62:7269-7281. 10.1080/10408398.2021.1913399 33938774

[38] Stevenson L Phillips F O’Sullivan K Walton J. Wheat bran: its composition and benefits to health, a European perspective. *Int J Food Sci Nutr.* 2012;63:1001-1013. 10.3109/09637486.2012.687366 22716911 PMC3507301

[39] Shewry P. The Contribution of Wheat to Human Nutrition and Health. A year on the field. Hoboken, NJ: Wiley;2021

[40] Burgess-Champoux T Marquart L Vickers Z Reicks M. Perceptions of children, parents, and teachers regarding whole-grain foods, and implications for a school-based intervention. *J Nutr Educ Behav.* 2006;38:230-237. 10.1016/j.jneb.2006.04.147 16785092

[41] Marquart L Pham A Lautenschlager L Croy M Sobal J. Beliefs about whole-grain foods by food and nutrition professionals, health club members, and special supplemental nutrition program for women, infants, and children participants/State fair attendees. *J Am Diet Assoc.* 2006;106:1856-1860. 10.1016/j.jada.2006.08.005 17081837

[42] McMackin E Dean M Woodside J McKinley M. Whole grains and health: attitudes to whole grains against a prevailing background of increased marketing and promotion. *Public Health Nutr.* 2013;16:743-751. 10.1017/S1368980012003205 23102407 PMC10271380

[43] Foster S Beck E Hughes J Grafenauer S. Whole Grains and Consumer Understanding: investigating Consumers’ Identification, Knowledge and Attitudes to Whole Grains. *Nutrients.* 2020;12:2170. 10.3390/nu12082170 32708000 PMC7468996

[44] Barrett E Foster S Beck E. Whole grain and high-fibre grain foods: how do knowledge, perceptions and attitudes affect food choice? *Appetite.* 2020;149:104630. 10.1016/j.appet.2020.104630 32057840

[45] Zanini B Simonetto A Marconi S Marullo M Castellano M Gilioli G. Whole grain perceptions and consumption attitudes: Results of a survey in Italy. *Health Educ J.* 2023;82:739-751.

[46] Kuznesof S Brownlee I Moore C Richardson D Jebb S Seal C. WHOLEheart study participant acceptance of wholegrain foods. *Appetite.* 2012;59:187-193. 10.1016/j.appet.2012.04.014 22546716

[47] Pohjanheimo T Luomala H Tahvonen R. Finnish adolescents’ attitudes towards wholegrain bread and healthiness. *J Sci Food Agric.* 2010;90:1538-1544. 10.1002/jsfa.3982 20549809

[48] Meynier A Chanson-Rollé A Riou E. Main Factors Influencing Whole Grain Consumption in Children and Adults-A Narrative Review. *Nutrients.* 2020;12:2217. 10.3390/nu12082217 32722381 PMC7468875

[49] Suthers R Broom M Beck E. Key Characteristics of Public Health Interventions Aimed at Increasing Whole Grain Intake: a Systematic Review. *J Nutr Educ Behav.* 2018;50:813-823. 10.1016/j.jneb.2018.05.013 30076022

[50] Moher D Shamseer L Clarke M Ghersi D Liberati A Petticrew M Preferred reporting items for systematic review and meta-analysis protocols (PRISMA-P) 2015 statement. *Syst Rev.* 2015;4:1. 10.1186/2046-4053-4-1 25554246 PMC4320440

[51] Kocher M Schulz R Lundquist H Peterson H Marquart L. Exploring US consumer grain consumption patterns: a scoping review protocol. Charlottesville: OSF. 2024.

[52] Peters M G McInerney P Munn Z Tricco A Khalil H. Scoping reviews 2020. 2024 edition. ed. Adelaide: JBI Manual for Evidence Synthesis; 2024.

[53] Tricco A Lillie E Zarin W O’Brien K Colquhoun H Levac D PRISMA Extension for Scoping Reviews (PRISMA-ScR): checklist and Explanation. *Ann Intern Med.* 2018;169:467-473. 10.7326/M18-0850 30178033

[54] Chen P Antonelli M. Conceptual Models of Food Choice: influential Factors Related to *Foods*, Individual Differences, and Society. *Foods.* 2020;9:1898. 10.3390/foods9121898 33353240 PMC7766596

[55] Hoffmann N Symmank C Mai R Stok F Rohm H Hoffmann S. The influence of extrinsic product attributes on consumers’ food decisions: review and network analysis of the marketing literature. *J Marketing Manag.* 2020;36:888-915. 10.1080/0267257X.2020.1773514

[56] Lu P Parrella JA Xu Z Kogut A. A scoping review of the literature examining consumer acceptance of upcycled foods. *Food Qual Prefer.* (2024) 114:105098. 10.1016/j.foodqual.2023.105098

[57] Setiono F Gangrade N Leak TM. U.S. Adolescents’ Diet Consumption Patterns Differ between Grocery and Convenience Stores: national Health and Nutrition Examination Survey 2011-2018. *Int J Environ Res Public Health.* 2021;18:8474. 10.3390/ijerph18168474 34444223 PMC8394683

[58] Taylor C Spees C Markwordt A Watowicz R Clark J Hooker N. Differences in US Adult Dietary Patterns by Food Security Status. *J Consumer Affairs.* 2017;51:549-565. 10.1111/joca.12166

[59] Lin W Nayga R Yang W. Preferences and willingness to pay for a novel carbon label: A choice experiment in the United States. *J Agric Appl Econ Assoc.* 2024;3:346-357. 10.1002/jaa2.116

[60] Combest S Warren C. Perceptions of college students in consuming whole grain foods made with Brewers’ Spent Grain. *Food Sci Nutr.* 2019;7:225-237. 10.1002/fsn3.872 30680176 PMC6341135

[61] Hicks-Roof K Arikawa A Schwam M Osorio D Walker K. College students approve: Using sorghum to increase whole-grain exposure in university foodservice. *J Foodservice Bus Res.* 2021;24:62-73. 10.1080/15378020.2020.1844526

[62] Wongprawmas R Sogari G Menozzi D Pellegrini N Lefebvre M Gómez M Determinants of US University Students’ Willingness to Include Whole Grain Pasta in Their Diet. *Int J Environ Res Public Health.* 2021;18:3173. 10.3390/ijerph18063173 33808575 PMC8003352

[63] Dominick S Bir C Widmar N Acharya L Wang H Wilcox M. Exploring preferences beyond the (cereal) box: ready-to-eat breakfast cereal buying behaviors. *Int Food Agribusiness Manag Rev.* 2018;21:1185-1202. 10.22434/IFAMR2017.0113

[64] Samuel L Wimmer I Glassberg G Boyar A. Familiarization with White Whole Wheat Improves Acceptability of Whole Grain-Based Baked Products Made with White Whole Wheat Among College Students. *Am J Lifestyle Med.* 2022;19:155982762211317. 10.1177/15598276221131763 40548238 PMC12177829

[65] Johnson T Mitchell-McCallister D Xu W. Consumer preference regarding a new corn variety: a willingness to pay study. *J Food Distribution Res.* 2024;55:46-64.

[66] Nicklas T Jahns L Bogle M Chester D Giovanni M Klurfeld D Barriers and facilitators for consumer adherence to the dietary guidelines for Americans: the HEALTH study. *J Acad Nutr Diet.* 2013;113:1317-1331. 10.1016/j.jand.2013.05.004 23871110

[67] Jahns L McDonald L Wadsworth A Morin C Liu Y Nicklas T. Barriers and facilitators to following the Dietary Guidelines for Americans reported by rural, Northern Plains American-Indian children. *Public Health Nutr.* 2015;18:482-489. 10.1017/S136898001400041X 24679830 PMC10271680

[68] Chea M Mobley A. Interpretation and Understanding of the Dietary Guidelines for Americans Consumer Messages Among Low-Income Adults. *J Am Coll Nutr.* 2020;39:63-71. 10.1080/07315724.2019.1610918 31084517

[69] Chea M Mobley A. Factors Associated with Identification and Consumption of Whole-Grain Foods in a Low-Income Population. *Curr Dev Nutr.* 2019;3:nzz064. 10.1093/cdn/nzz064 31231712 PMC6581827

[70] Magalis R Giovanni M Silliman K. Whole grain foods: is sensory liking related to knowledge, attitude, or intake? *Nutr Food Sci.* 2016;46:488-503. 10.1108/NFS-09-2015-0101

[71] Tritt A Reicks M Marquart L. Reformulation of pizza crust in restaurants may increase whole-grain intake among children. *Public Health Nutr.* 2015;18:1407-1411. 10.1017/S1368980014001724 25157427 PMC10271276

[72] De Leon A Burnett D Rust B Casperson S Horn W Keim N. Liking and Acceptability of Whole Grains Increases with a 6-Week Exposure but Preferences for Foods Varying in Taste and Fat Content Are Not Altered: a Randomized Controlled Trial. *Curr Dev Nutr.* 2020;4:nzaa023. 10.1093/cdn/nzaa023 32190809 PMC7066376

[73] De Leon A Burnett D Rust B Lyly M Keim N. Initial implicit association between whole grains and taste does not predict consumption of whole grains in low-whole grain consumers: a pilot randomized controlled trial. *Front Nutr.* 2024;11:1408256. 10.3389/fnut.2024.1408256 39403397 PMC11471685

[74] Drugova T Curtis K Akhundjanov S. Organic wheat products and consumer choice: a market segmentation analysis. *Br Food J.* 2020;122:2341-2358. 10.1108/BFJ-08-2019-0626

[75] Drugova T Curtis K. Do consumers care about organic when consuming speciality bakery products or is it all about indulgence? Br Food J. (2024) 126:3740-3755. 10.1108/BFJ-02-2024-0153

[76] Akaichi F Nayga R Jr. Nalley L. Are there trade-offs in valuation with respect to greenhouse gas emissions, origin and food miles attributes? *Eur Rev Agric Econ.* 2017;44:3-31. 10.1093/erae/jbw008

[77] Yao P Ozier A Brasseur K Robins S Adams C Bachar D. Food pantry nutrition education about whole grains and self-efficacy. *Family Consumer Sci Res J.* 2013;41:426-437. 10.1111/fcsr.12028

[78] Leak T Gangrade N Tester J. Facilitators and barriers to preparing and offering whole grains to children diagnosed with prediabetes: qualitative interviews with low-income caregivers. *BMC Public Health.* 2021;21:931. 10.1186/s12889-021-10915-5 34001074 PMC8127312

[79] MacNab L Davis K Francis S Violette C. Whole Grain Nutrition Education Program Improves Whole Grain Knowledge and Behaviors Among Community-Residing Older Adults. *J Nutr Gerontol Geriatr.* 2017;36:189-198. 10.1080/21551197.2017.1384424 29252144

[80] Akaichi F Nayga R Nalley L. Consumers’ preferences and willingness to pay for greenhouse gas emissions, origin and food miles attributes: evidence from experimental auctions. Land Economy, Environment and Society Working Paper Series - SRUC. 2016:49. Cupar: SRUC

[81] Dunford E Miles D Popkin B Ng S. Whole Grain and Refined Grains: an Examination of US Household Grocery Store Purchases. *J Nutr.* 2022;152:550-558. 10.1093/jn/nxab382 34718663 PMC8826838

[82] Tester J Leung C Leak T Laraia B. Recent Uptrend in Whole-Grain Intake Is Absent for Low-Income Adolescents, National Health and Nutrition Examination Survey, 2005-2012. *Prev Chronic Dis.* 2017;14:E55. 10.5888/pcd14.160540 28682743 PMC5510329

[83] Lenk K Caspi C Harnack L Laska M. Customer Characteristics and Shopping Patterns Associated with Healthy and Unhealthy Purchases at Small and Non-traditional Food Stores. *J Community Health.* 2018;43:70-78. 10.1007/s10900-017-0389-5 28616707 PMC5730500

[84] Curtis K Allen K Ward R. Food consumption, attitude, and behavioral change among CSA members: a northern Utah case study. *J Food Distribution Res.* 2015;46:3-16.

[85] Arts J English C Greene G Lofgren IE. A nutrition intervention to increase whole grain intake in college students. *Top Clin Nutr.* 2016;31:222-231. 10.1097/TIN.0000000000000075

[86] Baker B Russell J. Capturing a Value-Added Niche Market: Articulation of Local Organic Grain. *Am J Agric Econ.* 2017;99:532-545. 10.1093/ajae/aaw100

[87] Mars M. From bread we build community: Entrepreneurial leadership and the co-creation of local food businesses and systems. *J Agric Food Syst Community Development.* 2016;5(3):63–77. 10.5304/jafscd.2024.133.029

[88] Meyer N Sacchi G Sartori C Fischer C. Establishing alternative grain networks: A comparison of case experiences in South Tyrol, Italy, and Colorado, United States. *J Agric Food Syst Community Development.* 2024;13:337-361

[89] GrowNYC. About Greenmarket’s Regional Grains Project 2016. New York, NY: GrowNYC

[90] Mars M. From bread we build community: Entrepreneurial leadership and the co-creation of local food businesses and systems. *J Agric Food Syst Community Development.* 2015;5:63-77. 10.5304/jafscd.2015.053.005

[91] Sluyter C LeBlanc K Hicks-Roof K. Boosting Whole-Grain Utilization in the Consumer Market: a Case Study of the Oldways Whole Grains Council’s Stamped Product Database. *Nutrients.* 2022;14:713. 10.3390/nu14030713 35277072 PMC8839132

[92] Heiniö R Liukkonen K Myllymäki O Pihlava J Adlercreutz H Heinonen S Quantities of phenolic compounds and their impacts on the perceived flavour attributes of rye grain. *J Cereal Sci.* 2008;47:566-575. 10.1016/j.jcs.2007.06.018

[93] Heiniö R Noort M Katina K Alam S Sozer N de Kock H Sensory characteristics of wholegrain and bran-rich cereal foods – A review. *Trends Food Sci Technol.* 2016;47:25-38. 10.1016/j.tifs.2015.11.002

[94] Kamar M Evans C Hugh-Jones S. Factors influencing adolescent whole grain intake: a theory-based qualitative study. *Appetite.* 2016;101:125-133. 10.1016/j.appet.2016.02.154 26921485

[95] West R Seetharaman K Duizer L. Effect of drying profile and whole grain content on flavour and texture of pasta. *J Cereal Sci.* 2013;58:82-88. 10.1016/j.jcs.2013.03.018

[96] Heilman C Lakishyk K Radas S. An empirical investigation of in-store sampling promotions. *Br Food J.* 2011;113:1252-1266. 10.1108/00070701111177674

[97] Neo J Brownlee I. Wholegrain Food Acceptance in Young Singaporean Adults. *Nutrients.* 2017;9:371. 10.3390/nu9040371 28397752 PMC5409710

[98] OTA. Growth of U.S. Organic Marketplace Accelerated in 2024. Capitol Street NW: Organic Trade Association; 2025

[99] Rosen R Burgess-Champoux T Marquart L Reicks M. Associations between whole-grain intake, psychosocial variables, and home availability among elementary school children. *J Nutr Educ Behav.* 2012;44:628-633. 10.1016/j.jneb.2010.02.007 21333603 PMC3129399

[100] Gittelsohn J Davis S Steckler A Ethelbah B Clay T Metcalfe L Pathways: lessons learned and future directions for school-based interventions among American Indians. *Prev Med.* 2003;37:S107-S112. 10.1016/j.ypmed.2003.08.001 14636815 PMC4854572

[101] Gittelsohn J Toporoff E Story M Evans M Anliker J Davis S Food Perceptions and Dietary Behavior of American-Indian Children, Their Caregivers, and Educators: formative Assessment Findings from Pathways. *J Nutr Educ.* 2000;32:2-13. 10.1016/s0022-3182(00)70504-8 27667857 PMC5033233

[102] WSU. WSU Breadlab About US; 2025. Pullman: WSU

[103] Price E Kissock K Barrett E Batterham M Beck E. Whole-grain modified Nova ultra-processed food definitions: a cross-sectional analysis of the impact on cardiometabolic risk measures when excluding high whole-grain foods from the ultra-processed food category in Australia. *Br J Nutr.* 2025;133;346-356. 10.1017/S0007114524002952 39710615

[104] Price E Du M McKeown N Batterham M Beck E. Excluding whole grain-containing foods from the Nova ultraprocessed food category: a cross-sectional analysis of the impact on associations with cardiometabolic risk measures. *Am J Clin Nutr.* 2024;119:1133-1142. 10.1016/j.ajcnut.2024.02.017 38417577

[105] Sluyter C Neess R Grafenauer S. Leveraging partnerships to amplify whole grain messaging. *J Cereal Sci.* 2022;105:103456. 10.1016/j.jcs.2022.103456

[106] Fuldkornpartnerskabet. The Whole Grain Logo Manual; 2021. Available online at: https://fuldkorn.dk/wp-content/uploads/2021/12/Fuldkornslogomanualen_revideret-udgave_gaeldende-fra-9.-juni-2021-31.-december-2022_English.pdf (accessed March 12, 2025).

[107] FullkornsFrämjandet. Choose more whole grains; 2025. FullkornsFrämjandet

[108] WGI. 2025 Whole Grain Declaration of Acts A Call to Build a Whole Grain Generation. Whole Grains Initative; 2025.

[109] Peters M Marnie C Colquhoun H Garritty C Hempel S Horsley T Scoping reviews: reinforcing and advancing the methodology and application. *Syst Rev.* 2021;10:263. 10.1186/s13643-021-01821-3 34625095 PMC8499488

[110] Du M Mozaffarian D Wong J Pomeranz J Wilde P Zhang F. Whole-grain food intake among US adults, based on different definitions of whole-grain foods, NHANES 2003-2018. *Am J Clin Nutr.* 2022;116:1704-1714. 10.1093/ajcn/nqac267 36446403 PMC9761734

[111] Maughan C Belletti G Marescotti A Balázs B Lazányi O Mengoni M. Against the grain: a commons approach to the governance of ‘Alternative Grain Networks’. *Agric Food Econ.* 2025;13:9. 10.1186/s40100-025-00352-y

[112] Hergesheimer C Wittman H. Weaving Chains of Grain: Alternative Grain Networks and Social Value in British Columbia. *Food Cult Soc.* 2012;15:375-393. 10.2752/175174412X13276629245803

[113] Forrest N Wiek A. Growing a sustainable local grain economy in Arizona: A multidimensional analytical case study of an alternative food network. *J Agric Food Syst Community Development.* 2021;10:1-22. 10.5304/jafscd.2021.102.031

[114] Stefani G Nocella G Sacchi G. Piloting a Meta-Database of Agroecological Transitions: An Example from Sustainable Cereal Food Systems. *Agriculture.* 2020;10:219. 10.3390/agriculture10060219

[115] Stedden C Silva E Ryan M Mallory E Darby H Dawson J Organic small grain production in the Upper Midwest and Northeast: Challenges and lessons learned. *Agron J.* 2024;114:2735-2753. 10.1002/agj2.21658

[116] Hills K. Improving baking quality and local markets for wheat grown in western. Washington: Washington State University; 2012.

[117] Hills K Goldberger J Jones S. Commercial Bakers’ View on the Meaning of “Local” Wheat and Flour in Western Washington State. *J Agric Food Syst Community Development.* 2013;3:1-20. 10.5304/jafscd.2013.033.022

[118] Hills K Goldberger J Jones S. Commercial bakers and the relocalization of wheat in western Washington State. *Agric Human Values.* 2013;30:365-378. 10.1007/s10460-012-9403-9

[119] Carslisle L. Flour Power. YES! Magazine 2019.

[120] Stevenson S Lev L. Values-based food supply chains: Shepherd’s Grain. Online Source. 2013.

[121] Peterson H Feenstra G Ostrom M Tanaka K Brekken C Engelskirchen G. The value of values-based supply chains: farmer perspective. *Agric Human Values.* 2022;39:385-403. 10.1007/s10460-021-10255-5 34456465 PMC8382098

[122] Kissing Kucek L Dyck E Russell J Clark L Hamelman J Burns-Leader S Evaluation of wheat and emmer varieties for artisanal baking, pasta making, and sensory quality. *J Cereal Sci.* 2017;74:19-27. 10.1016/j.jcs.2016.12.010

[123] Galli F Smith J Brunori G Barling D. Global, Regional and Local food chains: an assessment of sustainability performance of wheat-to-bread chains across Italy and the UK. Conference: Agriculture in an Urbanizing Society. Reconnecting Agriculture and Food Chains to Societal Needs. 2015. San Francisco, CA: Wordpress

[124] Galli F Bartolini F Brunori G Colombo L Gava O Grando S Sustainability assessment of food supply chains: an application to local and global bread in Italy. *Agric Food Econ.* 2015;3:1-17.

[125] Walton S. Transforming the food system in ‘unprotected space’: the case of diverse grain networks in England. *Agric Human Values.* 2024;41:989-1006. 10.1007/s10460-023-10535-2 39183775 PMC11341705

[126] Guptill A Wilkins J. Buying into the food system: Trends in food retailing in the US and implications for local foods. *Agric Hum Values.* 2002;19:39-51. 10.1023/A:1015024827047

[127] Johnston S. From Farm to Bakery: Building Value Chains for Regionally-Grown and Milled Grains. New York, NY: GrowNYC: 2012.

[128] GrowNYC. GrowNYC Grainstand Evaluation. New York, NY: CUNY Urban Food Policy Institute; 2022.

[129] CGE. The Mid-Atlantic Grains Program: A Case Study. Washington, DC: Common Grain Alliance FRESHFARM; 2024.

[130] Asprooth L M. Hartman A. McFarland A. Galt R. Prokopy L. Drivers and deterrents of small grain adoption in the Upper Midwest. Wisconsin: Michael Fields Institute. 2023.

[131] DeLonge M Miles A Carlisle L. Investing in the transition to sustainable agriculture. *Environ Sci Policy.* 2016;55:266-273. 10.1016/j.envsci.2015.09.013

[132] NewYorkState. State Liquor Authority Celebrates Growth of Craft Beverage Industry on 5th Anniversary of the Craft New York Act 2025. Available online at: https://sla.ny.gov/news/state-liquor-authority-celebrates-growth-craft-beverage-industry-5th-anniversary-craft-new

[133] USDA. USDA Farm Storage Facility Loan Program; 2025. Washington, DC: USDA

[134] USDA. Resilient Food Systems Infrastructure Program; 2025. Washington, DC: USDA

[135] Kavet R. The economic impact of craft brewing on the Vermont economy in 2014. 2015. Available online at: https://legislature.vermont.gov/Documents/2018/WorkGroups/Senate%20Economic%20Development/Agencies%20and%20Organizations/Vermont%20Brewers%20Association/W~Sean%20Lawson~Economic%20Study%20-%20VT%20Brewers%20Assoc.~1-11-2017.pdf

[136] Halloran A. Neighbor Loaves program aims to maintain regional grain value chains and feed the community. *J Agric Food Syst Community Dev.* 2020;9:41-44. 10.5304/jafscd.2020.093.031

[137] USDA. Farmer’s Markets Accepting SNAP benefits; 2025. Washington, DC: USDA

[138] Network FF. Double Up Food Bucks 2021. Available online at: https://doubleupamerica.org

[139] WGC. 2025 Local Grains Consumer Insights Survey. 2025. WGC: Boston, MA

[140] Gosine L McSweeney M. Consumers’ attitudes towards alternative grains: a conjoint analysis study. *Int J Food Sci Technol.* 2019;54:1588-1596. 10.1111/ijfs.14126

[141] Wendin K Mustafa A Ortman T Gerhardt K. Consumer Awareness, Attitudes and Preferences Towards Heritage Cereals. *Foods.* 2020;9:742. 10.3390/foods9060742 32512763 PMC7353474

[142] Teuber R Dolgopolova I Nordstrom J. Some like it organic, some like it purple and some like it ancient: Consumer preferences and WTP for value-added attributes in whole grain bread. *Food Quality Preference.* 2016;52:244-254. 10.1016/j.foodqual.2016.05.002

[143] Shah P Dhir A Joshi R Tripathy N. Drivers and barriers in the consumption of alternative staples. A systematic literature review and future research agenda. *Br Food J.* 2021;123:3726-3759. 10.1108/BFJ-12-2020-1098

[144] WGC. Whole Grain Council Consumer Insights Survey. 2025. WGC: Boston, MA

[145] Mills A. By the numbers: Growth for Ancient Grains. 2023.

[146] Onufrak S Zaganjor H Moore L Hamner H Kimmons J Maynard L Foods Consumed by US Adults From Cafeterias and Vending Machines: nhanes 2005 to 2014. *Am J Health Promot.* 2019;33:666-674. 10.1177/0890117118821846 30602283 PMC6812485

