# Supplementary material for: U.S. consumer acceptance of whole grains, local grains, and alternative grain networks: a scoping review
Source: Front Nutr. 2026 Feb 13;13:1729260. doi: 10.3389/fnut.2026.1729260 (PMC12947702; doi:10.3389/fnut.2026.1729260)
Supplement: Supplementary file 1 [file Table_1.docx]

# Supplementary Material: Search Strategies

### CAB Abstracts

1 (bread* or grain* or millet* or wheat* or barley or maize or oats or rice or rye or sorghum or triticale or buckwheat* or corn or sonora or kamut or kernza or emmer or farro or spelt or amaranth or quinoa or legumes or pseudocereal*).ti,ab.

2 exp bread/ or grain/ or exp cereals/ or cereal grains/ or exp food grains/

3 1 or 2

4 consumer*.ti,ab.

5 consumers/

6 4 or 5

7 (((diet* or food or consumer* or grain*) adj3 (accept* or attitude* or behavior* or behaviour* or consumption or education or expenditure* or choice or purchas* or habit or liking or motivat* or perception* or preference* or spending)) or flavor or flavour or calor* intake).ti,ab.

8 attitudes/ or awareness/ or consumer attitudes/ or consumer behaviour/ or consumer education/ or consumer expenditure/ or consumer information/ or consumer preferences/ or consumer satisfaction/ or consumer surveys/ or demand/ or eating patterns/ or food consumption/ or food intake/ or food preferences/ or food marketing/ or food purchasing/ or health promotion/ or perception/ or taste/ or trends/ or exp nutrition/ or prices/ or food prices/ or calories/ or caloric intake/

9 7 or 8

10 3 and 6 and 9

11 limit 10 to english language

12 limit 11 to yr="2013 -Current"

### Agricola

1 (bread* or grain* or millet* or wheat* or barley or maize or oats or rice or rye or sorghum or triticale or buckwheat* or corn or sonora or kamut or kernza or emmer or farro or spelt or amaranth or quinoa or legumes or pseudocereal*).ti,ab.

2 exp breads/ or grains/ or exp food grains/ or exp small cereal grains/

3 1 or 2

4 consumer*.ti,ab.

5 "consumers (people)"/

6 4 or 5

7 (((diet* or food or consumer* or grain*) adj3 (accept* or attitude* or behavior* or behaviour* or consumption or education or expenditure* or choice or purchas* or habit or liking or motivat* or perception* or preference* or spending)) or flavor or flavour or calor* intake).ti,ab.

8 “attitudes and opinions”/ or exp consumer behavior/ or consumer education/ or exp consumer expenditure/ or consumer information/ or exp consumer surveys/ or diet/ or eating habits/ or flavor/ or exp food choices/ or food consumption/ or food intake/ or food marketing/ or exp food purchasing/ or foodshed/ or grain consumption/ or exp health promotion/ or healthy diet/ or healthy eating habits/ or human nutrition/ or local food systems/ or taste/ or nutrition/ or prices/ or food prices/ or energy intake/

9 7 or 8

10 3 and 6 and 9

11 limit 10 to english language

12 limit 11 to yr="2013 -Current"

### Scopus

( TITLE-ABS ( bread* OR grain* OR millet* OR wheat* OR barley OR maize OR oats OR rice OR rye OR sorghum OR triticale OR buckwheat* OR corn OR sonora OR kamut OR kernza OR emmer OR farro OR spelt OR amaranth OR quinoa OR legumes OR pseudocereal* OR cereal* ) ) AND ( TITLE-ABS ( consumer* ) ) AND ( TITLE-ABS ( ( diet* OR food OR consumer* OR grain* ) W/2 ( accept* OR attitude* OR behavior* OR behaviour* OR consumption OR education OR expenditure* OR choice OR purchas* OR habit OR liking OR motivat* OR perception* OR preference* OR spending ) ) OR TITLE-ABS ( flavor OR flavour OR calor* AND intake ) ) AND PUBYEAR > 2012 AND PUBYEAR < 2026 AND ( LIMIT-TO ( LANGUAGE , "English" ) )

### EconLit

((TITLE(bread* OR grain* OR millet* OR wheat* OR barley OR maize OR oats OR rice OR rye OR sorghum OR triticale OR buckwheat* OR corn OR sonora OR kamut OR kernza OR emmer OR farro OR spelt OR amaranth OR quinoa OR legumes OR pseudocereal* OR cereal*) OR ABSTRACT(bread* OR grain* OR millet* OR wheat* OR barley OR maize OR oats OR rice OR rye OR sorghum OR triticale OR buckwheat* OR corn OR sonora OR kamut OR kernza OR emmer OR farro OR spelt OR amaranth OR quinoa OR legumes OR pseudocereal* OR cereal*)) AND (TITLE(consumer*) OR ABSTRACT(consumer*)) AND (TITLE((diet* OR food OR consumer* OR grain*) NEAR/2 (accept* OR attitude* OR behavior* OR behaviour* OR consumption OR education OR expenditure* OR choice OR purchas* OR habit OR liking OR motivat* OR perception* OR preference* OR spending) OR flavor OR flavour OR calor* AND intake) OR (ABSTRACT((diet* OR food OR consumer* OR grain*) NEAR/2 (accept* OR attitude* OR behavior* OR behaviour* OR consumption OR education OR expenditure* OR choice OR purchas* OR habit OR liking OR motivat* OR perception* OR preference* OR spending) OR flavor OR flavour OR calor* AND intake)))) AND la.exact("ENG") AND pd(2013-2025)

### Dissertations & Theses Global

((TITLE(bread* OR grain* OR millet* OR wheat* OR barley OR maize OR oats OR rice OR rye OR sorghum OR triticale OR buckwheat* OR corn OR sonora OR kamut OR kernza OR emmer OR farro OR spelt OR amaranth OR quinoa OR legumes OR pseudocereal* OR cereal*) OR ABSTRACT(bread* OR grain* OR millet* OR wheat* OR barley OR maize OR oats OR rice OR rye OR sorghum OR triticale OR buckwheat* OR corn OR sonora OR kamut OR kernza OR emmer OR farro OR spelt OR amaranth OR quinoa OR legumes OR pseudocereal* OR cereal*)) AND (TITLE(consumer*) OR ABSTRACT(consumer*)) AND (TITLE((diet* OR food OR consumer* OR grain*) NEAR/2 (accept* OR attitude* OR behavior* OR behaviour* OR consumption OR education OR expenditure* OR choice OR purchas* OR habit OR liking OR motivat* OR perception* OR preference* OR spending) OR flavor OR flavour OR calor* AND intake) OR (ABSTRACT((diet* OR food OR consumer* OR grain*) NEAR/2 (accept* OR attitude* OR behavior* OR behaviour* OR consumption OR education OR expenditure* OR choice OR purchas* OR habit OR liking OR motivat* OR perception* OR preference* OR spending) OR flavor OR flavour OR calor* AND intake)))) AND la.exact("ENG") AND pd(2013-2025)
